# Spatial Segregation in Eastern North Pacific Skate Assemblages

**DOI:** 10.1371/journal.pone.0109907

**Published:** 2014-10-20

**Authors:** Joseph J. Bizzarro, Kristin M. Broms, Miles G. Logsdon, David A. Ebert, Mary M. Yoklavich, Linda A. Kuhnz, Adam P. Summers

**Affiliations:** 1 School of Aquatic and Fishery Sciences, University of Washington, Seattle, Washington, United States of America; 2 Cooperative Fish and Wildlife Unit, Colorado State University, Fort Collins, Colorado, United States of America; 3 School of Oceanography, University of Washington, Seattle, Washington, United States of America; 4 Pacific Shark Research Center, Moss Landing Marine Laboratories, Moss Landing, California, United States of America; 5 National Marine Fisheries Service–Southwest Fisheries Science Center–Fisheries Ecology Division, Santa Cruz, California, United States of America; 6 Monterey Bay Aquarium Research Institute, Moss Landing, California, United States of America; 7 Friday Harbor Laboratories, University of Washington, Friday Harbor, Washington, United States of America; Aristotle University of Thessaloniki, Greece

## Abstract

Skates (Rajiformes: Rajoidei) are common mesopredators in marine benthic communities. The spatial associations of individual species and the structure of assemblages are of considerable importance for effective monitoring and management of exploited skate populations. This study investigated the spatial associations of eastern North Pacific (ENP) skates in continental shelf and upper continental slope waters of two regions: central California and the western Gulf of Alaska. Long**-**term survey data were analyzed using GIS/spatial analysis techniques and regression models to determine distribution (by depth, temperature, and latitude/longitude) and relative abundance of the dominant species in each region. Submersible video data were incorporated for California to facilitate habitat association analysis. We addressed three main questions: 1) Are there regions of differential importance to skates?, 2) Are ENP skate assemblages spatially segregated?, and 3) When skates co-occur, do they differ in size? Skate populations were highly clustered in both regions, on scales of 10s of kilometers; however, high-density regions (i.e., hot spots) were segregated among species. Skate densities and frequencies of occurrence were substantially lower in Alaska as compared to California. Although skates are generally found on soft sediment habitats, *Raja rhina* exhibited the strongest association with mixed substrates, and *R. stellulata* catches were greatest on rocky reefs. Size segregation was evident in regions where species overlapped substantially in geographic and depth distribution (e.g., *R. rhina* and *Bathyraja kincaidii* off California; *B. aleutica* and *B. interrupta* in the Gulf of Alaska). Spatial niche differentiation in skates appears to be more pronounced than previously reported.

## Introduction

Determining distribution and abundance patterns of sympatric organisms and the mechanisms driving these patterns are fundamental aspects of ecological research. Estimating species composition and relative abundance within an assemblage and a better understanding of ecosystem processes provide a scientific basis for effective monitoring and management of exploited marine fishes. For fishes that share a close association with the benthos, a detailed understanding of spatial associations can inform the designation of no-take zones or marine protected areas (MPAs). These tools are in widespread use to conserve and rebuild multiple sympatric fish stocks. For example, the density and mean length of four reef fishes were significantly greater in a no-take zone compared to adjacent fished regions in the southwestern Indian Ocean [1]. Off California, substantial recent advancements in the knowledge of rockfish (*Sebastes* spp.) habitat associations have been a driving force in the development of a spatial fishery management strategy that has led to the establishment of nearly 200 MPAs [2], [3]. Benefits of spatial closures are not restricted to reef fishes on rocky habitats. The establishment of a no-take zone for flatfishes in the Baltic Sea resulted in increased densities of two exploited species and a net larval export to fished areas [4].

Skates (Rajiformes: Rajidae) are represented by nearly 300 species of benthic, egg-laying, cartilaginous fishes that constitute one-quarter of all chondrichthyan species [5]. They are common meso- and upper-trophic level predators in temperate and boreal regions, at depths ranging from the intertidal to the abyss [6], [7], [8]. Skates are among the most common bycatch in groundfish fisheries, and have been subjected to periodic direct exploitation [9], [10]. Skates present a suite of difficulties for determining accurate distribution and abundance patterns. They are remarkably morphologically conservative with a narrow palette of coloration, and therefore are challenging to identify for all but the specifically trained observer. Their habit of concealing themselves beneath the substrate leads to generalized taxonomic designations (i.e., “unidentified skate”) or missed sightings on submersible transects. Finally, although skates are typically associated with soft bottom, some species are found on mixed sediments or rocky substrates, so they are not adequately sampled in bottom trawl surveys. Developing spatial models for skates will require a synthesis of sampling techniques and data sources, as well as careful verification of species for some sampling modalities.

Fishery exploitation has affected the abundance, distribution, and species composition of skates and skate assemblages. However, the impact of fishing on skate populations is variable in magnitude and direction, and cannot be precisely predicted because most skate life histories are poorly known. For example, substantial increases in the biomass of skates have been reported in some heavily exploited regions whereas declines have been noted in others [11], [12], [13], [14]. Conflicting estimated population trends of some skate stocks could be due to a poor understanding of distribution and spatial associations that lead to erroneous interpretation of survey or fishery catch rate data [15], [16], [17], [18]. Even when overall skate biomass trends appear to be stable, substantial population fluctuations may occur among individual skate stocks [19], [20], [21], [22], [23]. Where species specific data are available, the typical response of skate assemblages to exploitation has been a decline in the abundance of relatively large, long-lived species (some of which are endangered) and an inverse trend among small, short-lived species [24], [25], [26]. However, misidentification and taxonomic uncertainty are common problems that complicate understanding skate population dynamics [9], [27], [28], [29], [30], [31].

Recent spatial studies of skates suggest complex ontogenetic and seasonal dynamics, including aggregating behavior and shifts in local abundance in association with depth changes and periodic migrations [23], [32], [33]. Skates, like other elasmobranchs, are known to exhibit different distribution patterns throughout ontogeny, with juveniles often occurring in deeper (i.e., *Bathyraja* spp.) or shallower (i.e., *Raja* spp.) waters than egg cases or adults [32], [33], [34], [35]. Although historically considered to inhabit unconsolidated, soft-bottom habitats, recent video data has indicated that several species are primarily associated with rocky substrates, including high-relief regions (Kuhnz et al., unpublished data). In addition to variable substrate associations, sympatric skate species are known to exhibit distinct depth zonation which limits their spatial overlap [36]. This depth zonation may actually be a function of variable temperature preferences among species, as temperature appears to be a key factor in determining distribution patterns and seasonal movements [16], [37]. Abundance and diversity of skates tend to reach a peak at the continental shelf break, where the shallower dwelling *Raja* species and deeper *Bathyraja* species typically overlap [36], [38]. Skates are often spatially aggregated, and the composition of these aggregations may temporally vary by sex and life stage [23], [37]. Migration patterns are poorly understood, but most appear to coincide with seasonal movements to breeding grounds and/or egg deposition sites [23].

Spatial studies of skates in the eastern North Pacific (ENP) have begun to clarify patterns of distribution and abundance among species. Five species dominate skate biomass in continental shelf and upper continental slope waters (≤600 m) off central California and in the Gulf of Alaska (GOA) [38], [39]. Two of these species, the big skate (*Beringraja binoculata*, formerly *Raja binoculata*; [40]) and longnose skate (*Raja rhina*), co-occur between regions. Spatial studies of skates off the U.S. West Coast consist of large-scale investigations of the composition of skate and demersal teleost assemblages [13], [41]. These studies demonstrated that *R. rhina* and, to a lesser extent, the sandpaper skate (*Bathyraja kincaidii*) are among the most abundant groundfish species by biomass, with peaks in abundance on the upper continental slope. Large-scale distribution and abundance patterns of skates in the GOA were recently described and indicated that *R. rhina* is the most abundant species by biomass, followed by *B. binoculata* [38]. Spatial segregation of skate species and assemblage composition was documented off southeastern Alaska [42]. In addition, nursery grounds of several skate species recently have been found in the Bering Sea, and suggest that skates regularly migrate to these areas to deposit their eggs [32], [43].

Recent U.S. management decisions and data needs highlight the importance of understanding spatial structure of skate stocks and guilds. A directed fishery developed for *B. binoculata* and *R. rhina* in 2003 off Kodiak Island but was considered unsustainable and curtailed in 2005 [10]. However, this fishery demonstrated that skate landings are lucrative, and retention of incidentally captured skates in the GOA has increased as a result [44]. Recognizing their variable life histories and vulnerability to exploitation, skates in the GOA were separated from a catch-all management group termed “Other Species” in 2005. Since then, separate fishing quotas have been established for the dominant, shallow water (<200 m) species, *B. binoculata* and *R. rhina*, whereas skates occurring in deeper water (e.g., Aleutian skate, *Bathyraja aleutica*, Bering skate, *B. interrupta*) or in low relative abundance (e.g., Alaska skate, *B. parmifera*) are managed as a complex [38]. Directed fishing has been prohibited for all skate species in federal waters of the GOA since 2005. A second directed fishery for *B. binoculata* and *R. rhina* was attempted in the state waters of Prince William Sound during 2009 and 2010 by the Alaska Department of Fish and Game (ADFG), but also was terminated because of a lack of profitability and observed declines in the landings of large females (K. Goldman, ADFG, Homer; pers. comm.). However, retention of incidental skate catch in regions such as Kodiak Island, Lower Cook Inlet, and Prince William Sound remains a management concern [10], [38]. Locating aggregation sites (or hot spots) for individual skate species and for the overall GOA skate assemblage can help determine likely areas of high bycatch, which can inform fishery management.

Off the U.S. West Coast, three skate species are included in federal groundfish management plans, *B. binoculata*, *R. rhina*, and the California skate, *R. inornata*. In contrast to Alaska, delineation of essential fish habitat (EFH) for individual species and associating species with habitat guilds are fundamental to groundfish management along the West Coast, typically through the designation of MPAs and other no**-**take zones [45]. However, skate landings along the West Coast historically have not been identified to species and misidentification of recently reported landings has further complicated an understanding of assemblage structure and stability [9]. Therefore, a baseline condition for West Coast skate assemblages must be established as a necessary first step toward spatial management.

Comprehensive, species specific spatial studies are needed to determine distribution patterns of ENP skates, and the structure of regional skate assemblages. Improvements in skate identification by fisheries scientists have enabled this type of research for skate assemblages off central California and in the western GOA. Our initial question of interest was (Q1): Are there areas within the broader study regions that are of differential importance to skate species and their life stages? This question was evaluated by: a) determining broad-scale (regional) patterns of distribution and abundance in both study areas, b) using regression analysis to predict geographic and depth preferences on regional and local (i.e., inshore waters of the west-central GOA, Monterey Bay) scales in both study areas, and c) assessing habitat associations across a variety of scales off central California. Collectively, these results enabled an investigation of potential inter**-** and intraspecific variation in spatial associations, framed by the following question (Q2): Is there spatial segregation among skate species and their life stages and, if so, at what scale(s)? Finally, we asked (Q3): If distributions of skate species within an assemblage overlap, do they differ in size (i.e., total length, TL)? This question was addressed by: a) developing a randomization test to evaluate observed vs. expected species richness among regional bottom trawl hauls, and b) comparing mean sizes of species that exhibited substantial spatial overlap in trawl landings.

In summary, the overall aim of this study was to use existing, fishery**-**independent data to determine and compare spatial associations of the primary species and their life stages in two ENP skate assemblages. The determination of geographic, depth, and substrate associations broadens the current understanding of skate ecology, enables comparisons of assemblage structure with other skate and benthic marine teleost assemblages, and allows us to make recommendations to improve management of ENP skates based on a greater understanding of distributional patterns. Investigations of spatial and size segregation also contribute to the broader body of such literature concerning benthic and demersal marine fishes.

## Methods

### Data Collection: Central California

**Figure 1:**
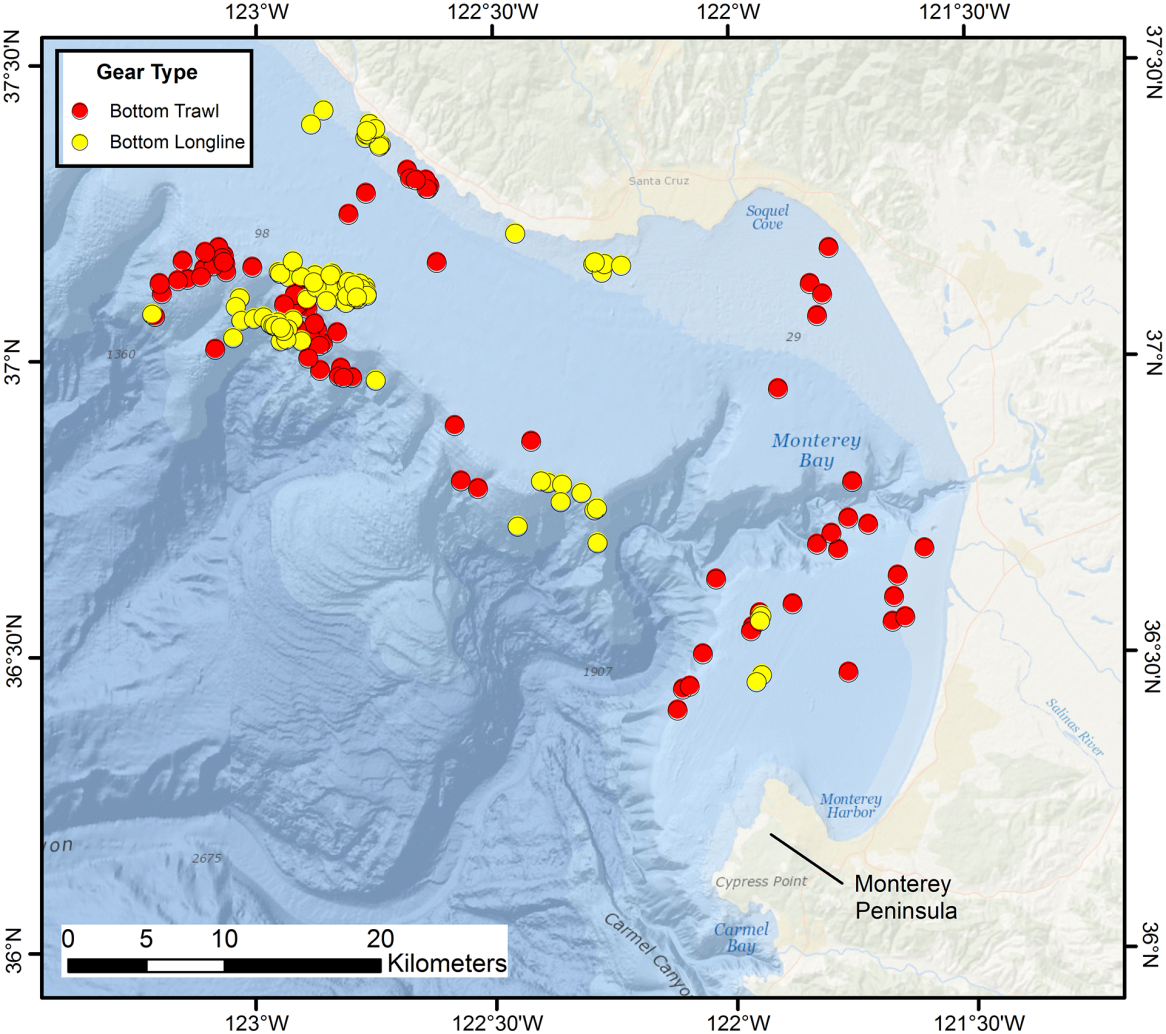
Trawl and longline sampling locations in Monterey Bay. National Marine Fisheries Service–Southwest Fisheries Science Center–Fisheries Ecology Division bottom set trawl (*n* = 80) and longline (*n* = 106) survey locations in the greater Monterey Bay region.

Data and specimens necessary for megascale (1–10s of km) spatial studies [46] of the five indicated skate species off central California were collected from bottom trawl and longline surveys conducted by regional offices of the U.S. National Marine Fisheries Service (NMFS) (Table 1). The inception of all data sets corresponds to the first year of reliable, species specific skate identifications, as determined by personal communication with survey personnel. The study site ranged from 36.0° N to 37.5° N, and corresponded to the spatial extent of published and ongoing life history and ecological research by the Pacific Shark Research Center (PSRC) (Moss Landing Marine Laboratories, California State University, Moss Landing, CA). The depth range extended from the shallowest sampled regions to the 600 m isobath. A truncated depth range was used for consistency with NMFS–Southwest Fisheries Science Center–Fisheries Ecology Division (SWFSC–FED) surveys conducted in the greater Monterey Bay area and to avoid overlap with a similar, deep-water study underway by Monterey Bay Aquarium Research Institute (MBARI) researchers in the same region. SWFSC–FED bottom trawl surveys targeted soft substrate regions, whereas bottom longline surveys were conducted on areas of extensive rock outcrop (Figure 1). All skates from these surveys were identified and sampled for biological information (e.g., TL, sex, maturity stage = juvenile, transitional, adult) by PSRC personnel. Maturity was determined using criteria established by [47]. Maturity status of skates was not determined for the coast-wide NMFS–Northwest Fisheries Science Center (NWFSC) and NMFS–Alaska Fisheries Science Center (AFSC) surveys that spanned the study range (Table 1). Instead, maturity of unsampled skates was assigned from TL data and corresponding length-at-maturity estimates, which were available for the skate populations within the study region [48], [49], [50], (Ebert, unpublished data).

**Table 1:** Details of sampling surveys conducted in the western Gulf of Alaska and off central California.

Region	Organization	Sampling Method	N	Month(s)	Year(s)	Depth Range	Median Depth	Depth Quartiles
						m	m	m
Central California	NMFS–AFSC*	Bottom Trawl	98	June-Oct	1998–2004	59–477	118	90–335
Central California	NMFS–AFSC**	Bottom Trawl	24	June-Oct	1997–2001	190–602	422	245–465
Central California	NMFS–NWFSC	Bottom Trawl	235	June-Oct	2003–2010	58–598	118	94–268
Monterey Bay	NMFS–SWFSC–FED	Bottom Longline	106	Jan-Dec	2002–2005	5–503	93	88–136
Monterey Bay	NMFS–SWFSC–FED	Bottom Trawl	80	Jan-Dec	2002–2003	9–614	144	66–294
Monterey Bay	MBARI	ROV	219	Jan-Dec	1997–2007	30–600	354	261–424
Monterey Bay	NMFS–SWFSC–FED	Manned Submersible	1203	Aug-Nov	1992–2009	24–316	107	73–193
Western GOA	NMFS–AFSC	Bottom Trawl	3931	May-Aug	1999–2011	13–984	121	80–180
Kamishak Bay	ADFG–Homer	Bottom Trawl	160	May-Aug	2003–2012	27–178	70	45–139
Alaska Peninsula	ADFG–Kodiak	Bottom Trawl	1368	July-Aug	2003–2012	10–115	55	45–72
Kodiak Island	ADFG–Kodiak	Bottom Trawl	1836	June-Sept	2003–2012	8–138	66	40–80
Shelikof Strait	ADFG–Kodiak	Bottom Trawl	278	Sept	2003–2012	32–158	85	65–100

Note: Data were collected yearly for the indicated ranges, with the following exceptions: NMFS–AFSC* = 1998, 2001, 2004; NMFS–AFSC** = 1997, 1999, 2001; NMFS–SWFSC–FED (submersible) = 1992–1994, 1997, 1998, 2002–2004, 2007–2009; western GOA = biennially; Kamishak Bay = 2003–2007, 2010, 2012.

* = effort was focused on continental shelf,

** = effort was focused on continental slope;

NMFS = National Marine Fisheries Service, AFSC = Alaska Fisheries Science Center, NWFSC = Northwest Fisheries Science Center, SWFSC–FED = Southwest Fisheries Science Center–Fisheries Ecology Division, MBARI = Monterey Bay Aquarium Research Institute, ADFG = Alaska Department of Fish and Game, ROV = remotely operated vehicle, N = number of samples for each method, Oct = October, Jan = January, Dec = December, Aug = August, Nov = November, Sept = September, m = meters.

Manned and unmanned submersible data were used to investigate meso- (10s of m to <1 km), macro- (1 to 10s of m), and microscale (<1 m) habitat associations [46] of the central California skate assemblage. All skates observed during manned submersible dives of the *Delta*, conducted by NMFS personnel, and unmanned, remotely operated vehicle (ROV) dives of the *Tiburon* and *Ventana*, conducted by MBARI personnel, were identified by the authors (Bizzarro, Ebert, and Kuhnz) from archived video (Table 1). Identifications were ranked (1 = positive identification, 2 = uncertain identification) and only positive, species-level identifications were utilized. Transect data from *Delta* dives were available from long-term groundfish habitat association studies conducted on the shelf and upper slope throughout the study region and facilitated quantitative analysis (Table 1). ROV dives extended to the 600 m limit, with the great majority occurring within the Monterey Bay Canyon System at depths >300 m (Table 1). Life stage was determined for ROV data, but too few observations were available to utilize maturity data for *Delta* dives. Whenever possible, lasers set at a fixed distance apart were used to estimate TL from ROV and manned submersible video data, which was compared to established length-at-maturity data [48], [49], [50], (Ebert, unpublished data). In some cases pelvic fins were clearly visible and the presence/absence and size of the paired copulatory organs (claspers) revealed an individual to be a mature or immature male or a female.

### Data Collection: Western Gulf of Alaska

**Figure 2:**
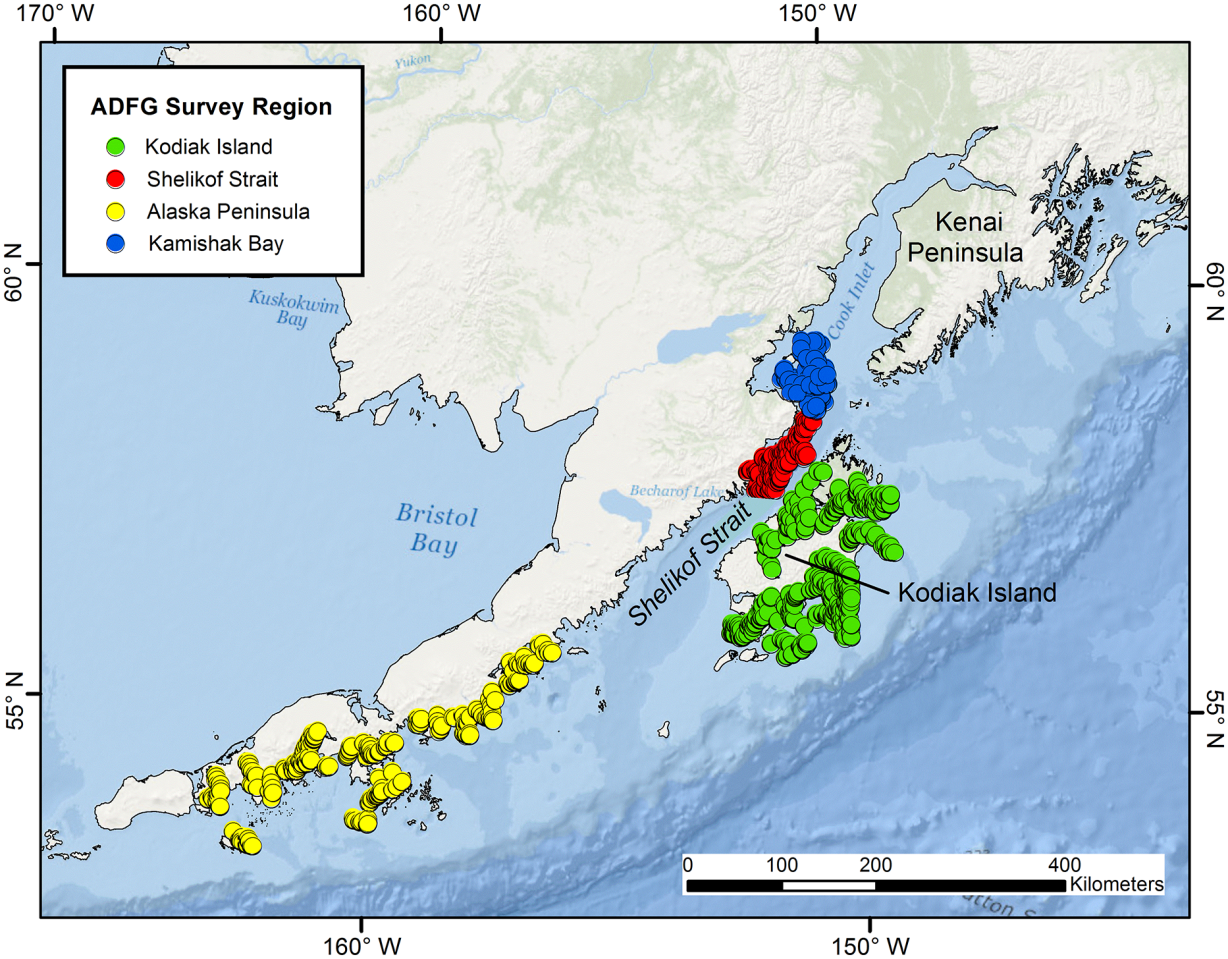
Trawl locations among regions in the west-central Gulf of Alaska. Alaska Department of Fish and Game trawl survey regions located within the larger study site in the western Gulf of Alaska. The total number of trawls conducted during 2003–2012 among regions was, as follows: Kodiak Island (*n* = 1836), Shelikof Strait (*n* = 278), Alaska Peninsula (*n* = 1368), and Kamishak Bay (*n* = 160).

Trawl data from NMFS–AFSC and ADFG were utilized for megascale spatial analysis in the western GOA. The study site was substantially larger than that off central California, ranging from 144.0° W to 164.0° W, but also corresponded to the spatial extent of published and ongoing PSRC life history and ecological research. Trawl sets were generally fished on soft substrate regions. Regional trawl surveys of ADFG, Kodiak (Alaska Peninsula, Kodiak Island, Shelikof Strait) and ADFG, Homer (Kamishak Bay) offices were conducted within the larger study site (Figure 2). NMFS trawls reached considerably deeper depths, but the majority of the effort for the NMFS and ADFG surveys was focused on the continental shelf (Table 1). Maturity stage of each species was estimated from TL data and corresponding length-at-maturity estimates for individuals collected within the study region [51], [52], [53], [54], or in close proximity [55].

### Spatial Analysis

Spatial analyses were conducted using NMFS–AFSC and NMFS–NWFSC bottom trawl data sets in California, and NMFS–AFSC data in the western GOA. Data sets encompassed the entirety of each study region, and were collected regularly over several years (Table 1). Spatial analyses addressed Q1 (i.e., areas of differential importance?) and Q2 (i.e., spatial segregation?) by determining and comparing the distribution and abundance patterns of species and their life stages, when possible.

Skate numerical and weight data, expressed as catch-per-unit-effort (#individuals/km^2^; kg/km^2^), were pooled across years to investigate long-term trends in abundance and biomass for every species in each skate assemblage. For this and all subsequent analyses, haul-specific mean depth data (taken from temperature-depth recorders or averaged from minimum and maximum depths) and gear midpoint locations (either provided or averaged from gear start and end points) were used whenever possible. However, only a starting location and depth were available for some of the analyzed hauls.

Spatial data analysis tools were used to link distribution and abundance patterns with the underlying process (or processes; e.g., depth, latitude) driving these patterns and the associated scale of the process. Autocorrelation and clustering were investigated on local (trends within the study region) and global (overall trend across the entire study region) scales [56]. All analyses were conducted in ArcMap v. 10.1 on data sets projected in UTM Zone 10N (WGS 1984) or Teale Albers (NAD 1927) for California and Alaska, respectively [57]. Incremental Spatial Autocorrelation (ISA), which uses Moran’s I measure to test for spatial autocorrelation across a series of distances throughout a pre-defined region (i.e., study site) [57], was conducted to determine the distance associated with peak clustering of skate biomass (e.g., ISA = peak clustering at 60 km). The value obtained from ISA was then used as the incrementing distance for iterative Multi-Distance Spatial Clustering (K-Function) [58], which assessed whether skate biomass exhibited statistically significant clustering or dispersion over a range of distances (e.g., K = significant clustering at a range of 40–100 km). Global spatial autocorrelation (Global Moran’s I) [59] was conducted to evaluate if skate biomass was clustered, dispersed, or randomly distributed within the overall study site. This measure ranges from −1 to 1, with values close to −1 indicating extreme dispersion, values close to 1 indicating extreme clustering, and values near zero indicating complete spatial randomness. High-Low Clustering (General G) [60], [61] was used to determine if there was significant spatial clustering of high or low values within the overall study region. The local version of the General G statistic (Gi*) was used to map and identify statistically significant regions of high (hot spots) and low (cold spots) values of skate abundance or biomass. I, G, and Gi* are indices that are used to generate an observed value for comparison with an expected value in order to evaluate statistical significance (p-value) and indicate the degree of autocorrelation or clustering (negative z-score = dispersed; positive z-score = clustered). Iterative randomization of CPUE values among the spatially fixed observational points (i.e., haul locations) was used to test the statistical significance of the observed pattern; therefore, the introduction of simulated locations was not required.

### Comparison of Size Distributions

Randomization tests were used to investigate species specific spatial segregation (Q2), and one-way ANOVA (with Tukey’s HSD post-hoc tests) and t-tests were used to determine if skate species that commonly occurred in trawls differed in size (Q3). The NMFS–NWFSC and NMFS–AFSC bottom trawl data sets were used for analysis off California and in the western GOA, respectively, and hauls with 0, 1, 2, 3, 4, or 5 skate species were recorded. For the randomization tests, an expected distribution of hauls containing 0–5 species was created to compare with the observed distribution. Because species occurrences were influenced by spatial location of hauls, binomial probabilities of occurrence were generated for each species in order to estimate the expected distribution. This step was accomplished by constructing generalized linear models with species richness (presence/absence) as the response variable and depth, latitude, and longitude as covariates. Akaike's information criterion was used to select the best models for each species. Binomial probability models for each species were combined and the expected distribution of species richness among hauls was randomized 999 times to form the expected distribution. Contingency tables with Chi-square statistics were used to determine if the observed species richness among hauls was significantly different than expected on an overall basis and, subsequently, for each level of species richness. Mean total length of species that commonly co-occurred in hauls was then compared to determine if size segregation was evident. Data for these comparisons were restricted to hauls in which both species were captured. Normality was assessed using histograms and residuals, and power analyses were conducted when significant differences were not found.

### Regression Models

Regression analysis was conducted using bottom trawl and bottom longline data sets to address Q1 and Q2, thereby complementing the previously described spatial analyses. Regression models were fit to data to determine variables that were associated with predicted numerical counts (response variable) of skate species and their life stages, and to estimate depth, geographic, and (indirectly) temperature ranges of common and maximum occurrence. Depth-temperature relationships for both regions were constructed using generalized additive models fit to data from the most expansive (global) data sets in each area, with depth as the response variable and temperature as the explanatory variable. Other covariates were not incorporated because model fits were robust, and so that established depth ranges could be isolated to predict temperature preferences directly.

The California NMFS-NWFSC data from 2003–2010 were modeled using zero-inflated Poisson (ZIP) regression, with effort (i.e., area swept) as an offset [62], because of an abundance of zero-catch data and dispersion issues with basic Poisson regression. The full model was fit for each sex-life stage of all species. Backwards model selection, with a cut-off of *p* = 0.05 was used to determine if regression coefficients significantly affected the distribution of a sex-life stage. The final life-stage specific models (without the inclusion of error terms) were then used to predict the magnitude of occurrence across the entire sampled depth and latitudinal ranges. The distribution of predicted maximum counts for sampled depth and latitude ranges were compared between transitional and adult males and females and combined if no significant covariates were found, or if the predicted depths and latitudes of maximum occurrence were within approximately 5% of the overall range of values. When individuals of a particular life stage were too few or too infrequently captured to construct robust models, transitional and adult males and females were first pooled, and then further combined with juveniles, if necessary. Covariates (depth, depth^2^, latitude, latitude^2^) were scaled to avoid computational problems in the optimization of regression models. Temperature was not included as a covariate because it was not consistently recorded and was highly correlated with depth. Longitude also was omitted because it was highly correlated with latitude (*ρ* = 0.91). Depth was log-transformed and the other covariates were scaled by their means and standard deviations. “Common” depth and geographic ranges were created as the locations where more than the median predicted counts were expected from the regression results. Regression model construction was generally consistent for SWFSC–FED trawl data, but oceanographic season (i.e., Davidson, December–February; Upwelling, March–July; Oceanic, August–November; after [63]) also was included, and tows were standardized by duration because no area-swept data were available.

Because skate catches were extremely low or absent in most western GOA AFSC trawl sets, these data were reduced to presence-absence and analyzed using logistic regression. Covariates included: depth, depth^2^, longitude, and longitude^2^ because skate distributional extents are generally oriented on longitudinal gradient in the GOA [38].

The ADFG data were analyzed using ZIP models, similar to the California data. However, length data were unavailable for the ADFG data set, precluding life-stage specific calculations. Numerical CPUE data were therefore analyzed with ZIP regression on a species specific basis. Covariates included: ADFG fishery management region, depth, and depth^2^.

ZIP models also were fit to the SWFSC–FED longline data for *R. stellulata*, and manned submersible data for *R. rhina*. All sex and age classes were combined for each species because low sample size precluded more detailed analysis. Longline data were not appropriate for analysis of other California skate species because they exhibited extremely low sample sizes relative to trawl data. Similarly, *R. rhina* was the only species for which sufficient manned submersible data were available to model distribution and habitat associations using multiple variables (mean depth, mean depth^2^, latitude, habitat type, area swept) and previously described regression techniques.

The MBARI and NMFS–AFSC data for California were not analyzed with regression models because of inherent deficiencies and biases. No estimates of area swept were available for MBARI ROV data, precluding quantitative analysis. The timing and design of NMFS–AFSC continental shelf and continental slope surveys were inconsistent with those more recently conducted by NMFS–NWFSC and therefore were not included.

### Habitat Association Analysis

Species specific determination of habitat associations was possible for the central California region, using the ROV and manned submersible data, to further investigate areas of differential importance (Q1) and potential spatial segregation (Q2) among skate species and their life stages. Analytical methods varied for *Delta*- and ROV-collected data sets because of inherent differences in their methods of data collection. SWFSC–FED personnel used the *Delta* to conduct quantitative, strip transects that could be used to determine densities of fishes among different habitat types, and thereby to facilitate spatial analysis (see [2], [64]). By contrast, ROV video data were collected opportunistically during a variety of seafloor operations, which varied substantially in their objectives and in the amount of seafloor surveyed. Therefore, these data could not be standardized to produce density estimates for skates, and were instead limited to presence-only data and treated qualitatively. ROV and manned submersible data were useful, however, in providing unambiguous depth records, observing behavior under natural conditions, and examining habitat characteristics. Habitat patches of specific substrate types and general composition within a *Delta* transect were collapsed to form three habitat categories for transects: 1) Hard (≥67% of area swept is rock), 2) Soft (≥67% of area swept is soft sediment), and 3) Mixed (<67% of area swept is rock and <67% of areas swept is soft sediment). Habitat preferences were then analyzed using Pearson’s chi-squared tests to compare observed and expected distributions of skates among transects of different habitat types, and an index of habitat electivity (summing to 1.0) was calculated to determine the relative magnitude of habitat preferences (after [65]).

ZIP regression model outputs were used to resolve inaccuracies in the spatial models used by NMFS to determine EFH for skates off the U.S. West Coast [66]. A habitat suitability probability (HSP) model was developed by NMFS and outside contractors to describe and identify EFH for each life stage of federally managed groundfishes [66]. HSP is a measure of the likelihood that a particular habitat (i.e., depth, latitude, and substrate type) is suitable for a fish species or life stage. This model requires prior knowledge or estimation of habitat suitability indices (HSIs) for depth (Z), latitude (Y), and substrate type (ST). HSP is then calculated from separate probabilities derived from HSIs for each habitat characteristic (Z, Y, ST) as HSP = HSI_yz_ * HSI_st_. Latitude and depth HSI (HSI_yz_) are combined and represented as a continuous variable (0–1) in the HSP model using binary presence/absence data. The midpoints of a species’ latitude and depth ranges were assigned a value of 1, and then a linear relationship was established between the midpoints and the spatial extents (i.e., endpoints) of each range. HIS_st_ was determined from the literature and given a value of 0, 0.33, 0.66, or 1.00, depending on perceived preference. Results are presented graphically as an HSP profile that spans the West Coast. Because only the static HSP profiles are available, the original HSP models were recreated following strict adherence to described procedures [66] to generate consistent maps for comparison with new model outputs.

The structure of the HSP model was retained to maintain consistency with the NMFS’ methods of determining EFH, but the inputs were improved to create more accurate model outputs. Minimum and maximum depth distributions were updated through a literature review, which resolved several inaccuracies. Preferred minimum and maximum depths were set to the common depth ranges established by NMFS−NWFSC ZIP models, as previously described. When available, the NMFS method used the 5^th^ and 95^th^ percentiles of the surveyed depth range as extents to establish preferred depths. However, such values were typically extrapolated from data rich to data poor species, such as skates. We used the optimal depths predicted with the ZIP models, instead of averaging them from preferred depth ranges. For both techniques, curves were fit through the five possible data points to generate suitability values (0–1). NMFS HSP models generate an optimal latitude by averaging latitude values at the preferred minimum and maximum depths. Because the study site was restricted to the central California coast, latitudinal distribution estimates were updated by averaging and scaling species specific CPUE estimates from 2003–2008 NMFS-NWFSC trawl surveys among five consecutive, non-overlapping regions that spanned the West Coast [67], and then fitting a curve through these five points. Distributional extents were updated, when possible, from a literature review and used to establish zero values. Updated substrate preference information was taken directly from preference indices established from manned submersible data (after [65]). Using these updated methods, a new HSP profile was created for transitional/adult specimens of *R. inornata* as an example, and compared to a recreation of the original model for this species.

## Results

### Central California Skate Assemblage

#### Spatial Analysis

Generally, skate distributions were clustered on a global scale (i.e., within the extents of the study region), significantly more than expected by chance. Global spatial autocorrelation (i.e., results of Global Moran’s I analyses) was most evident for *R. rhina* and *R. inornata*, with the distribution of numerical- and weight-specific CPUE values exhibiting highly significant spatial clustering (Table 2). The distribution of *B. kincaidii* was strongly clustered by abundance, but not by biomass (Table 2). Numerical and weight values for *B. binoculata* and *R. stellulata* were each globally clustered, but the complementary values were not significant (K; Table 2). All the hardnose skates (*Raja* and *Beringraja* spp.) exhibited significant high-low clustering (General G; Table 2) by abundance and/or biomass, with results driven by high-density aggregations. For *B. kincaidii*, spatial clustering of high abundance and biomass values displayed a significant departure from spatial randomness, but clustering was more pronounced in numerical catch data. When all skates were combined for analysis, global clustering was significant (Global Moran’s I; Table 2); but local clustering of high or low values was not (General G; Table 2).

**Table 2:** Spatial analysis results for the central California skate assemblage.

Species	N	WT	%FO	Measure	ISA	Global Moran's I	K	General G
					km		km	
*Beringraja binoculata*	73	504	10.0	CPUE (N)	15.00*	0.091**	NS	0.730*
				CPUE (WT)	23.04*	0.033	NS	0.712
*Raja rhina*	2787	4706	72.3	CPUE (N)	21.68*	0.086**	NS	0.028*
				CPUE (WT)	8.26*	0.082**	NS	0.032*
*Raja inornata*	697	465	28.0	CPUE (N)	13.63*	0.218**	52.25*	0.045**
				CPUE (WT)	13.63*	0.239**	51.28*	0.050**
*Raja stellulata*	30	40	3.1	CPUE (N)	16.31*	0.009	NS	0.056
				CPUE (WT)	16.31*	0.036*	NS	0.126*
*Bathyraja kincaidii*	1663	923	45.3	CPUE (N)	8.26*	0.238**	15.89*	0.050**
				CPUE (WT)	8.26*	0.005	18.83*	0.055*
All Species	5250	6,638	89.1	Species Richness	6.91*	0.069**	33.93*	0.023

NS = not significant. N = number, WT = weight (kilograms), %FO = frequency of occurrence among hauls, CPUE = catch-per-unit-effort (#/km^2^, kg/km^2^), ISA = incremental spatial autocorrelation, K = K Function, km = kilometers. Significant p-value thresholds are indicated as follows: * = <0.05, ** = <0.001. Data were collected during National Marine Fisheries Service, Northwest Fisheries Science Center and Alaska Fisheries Science Center trawl surveys (*n* = 422) conducted during 1997–2010 on the continental shelf and upper slope of central California.

Spatial processes promoting local clustering exhibited significant, maximum peaks for all species by both measures (CPUE N, CPUE WT), but the degree of clustering varied among species at broader spatial scales. ISA results indicated consistent values of maximum peak clustering between measures for *R. inornata*, *R. stellulata*, and *B. kincaidii*, as a result of consistent magnitudes between measures among fixed haul locations (Table 2). Maximum peak clustering occurred at scales ranging from 8.26 km (*B. kincaidii*) to 23.04 km (*B. binoculata*, CPUE WT) (ISA; Table 2). An opposite trend in max peak clustering was observed between *R. rhina* (greater distance by number) and *B. binoculata* (greater by weight) (ISA; Table 2). Although significant clustering was observed at specific distance intervals, broad-scale spatial clustering (K) was not significant for *R. rhina* or the two hardnose species with low sample sizes (*B. binoculata*, *R. stellulata*). The central California population of *R. inornata* was clustered at distances up to >50 km, whereas that of *B. kincaidii* was clustered over a considerably shorter distance range (K; Table 2). For both species, multi-distance spatial clustering results were consistent between measures (K; Table 2). When species were combined, significant peak clustering was observed at 6.91 km, and skates were clustered at distances of up to 33.93 km.

**Figure 3:**
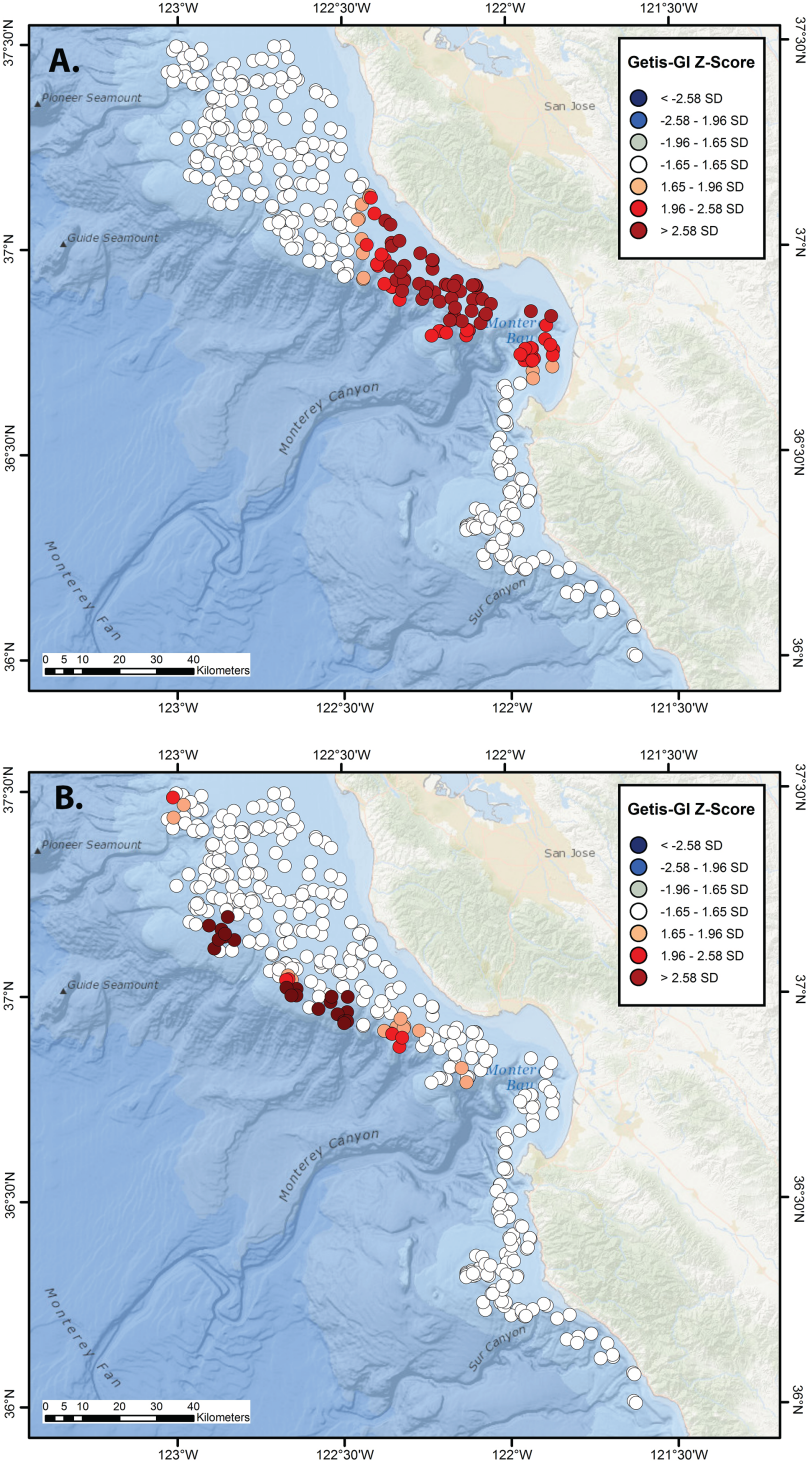
Hot and cold spot maps for *Beringraja binoculata* and *Raja rhina*. Getis-GI Hot Spot Analysis Z-score plots of catch-per-unit-effort (kg/km) for the hardnose skates, *Beringraja binoculata* (A) and *Raja rhina* (B), off central California, as calculated from NMFS–AFSC and NMFS–NWFSC trawl surveys conducted during 1997–2010.

**Figure 4:**
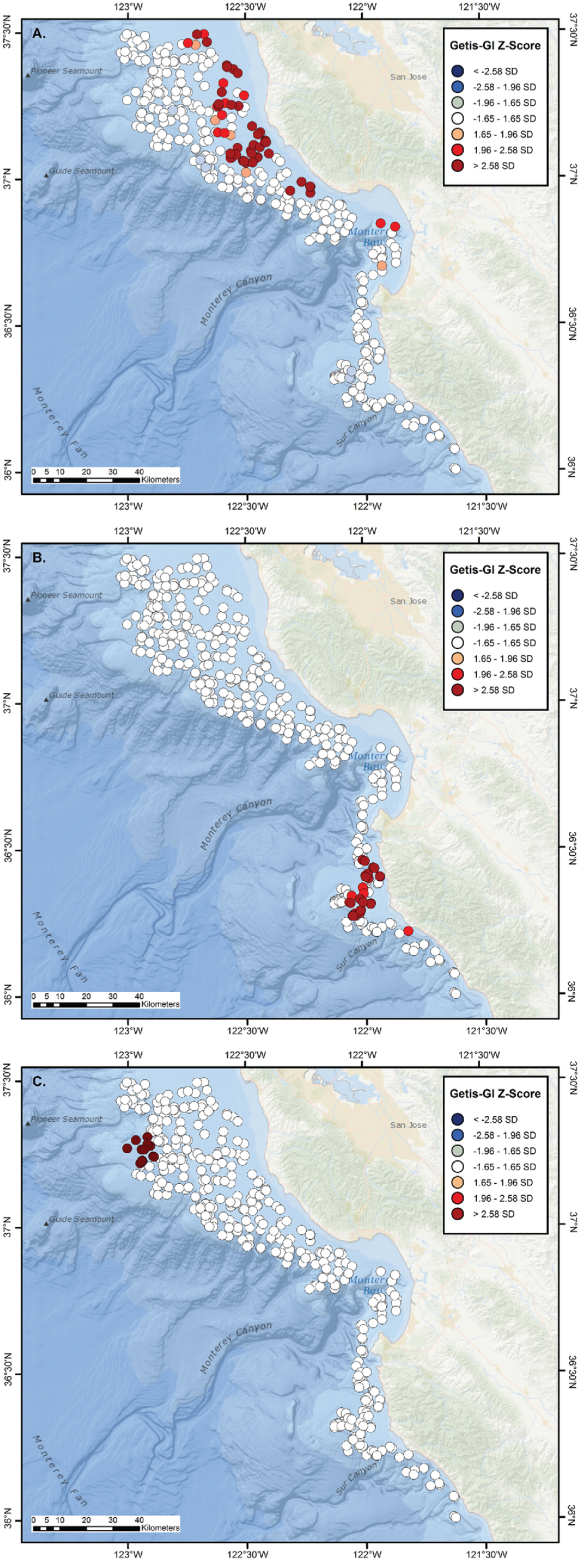
Hot and cold spot maps for *Raja inornata, R. stellulata, and Bathyraja kincaidii*. Getis-GI Hot Spot Analysis Z-score plots of catch-per-unit-effort (kg/km) for the California skate, *Raja inornata* (A); starry skate, *R. stellulata* (B), and sandpapaer skate, *Bathyraja kincaidii* (C) off central California, as calculated from NMFS–AFSC and NMFS–NWFSC trawl surveys conducted during 1997–2010.

Significant, largely distinct hot spots were observed for central California skate species, with clear cold spots also evident when overall skate distribution was analyzed. Significant aggregations of the two largest species, *B. binoculata* and *R. rhina*, were spatially segregated. The region of greatest *B. binoculata* clustering extended from south-central Monterey Bay northward to >37° N, with results of lower significance at the outer edge of the surveyed area (Getis-G z-scores; Figure 3a). Isolated aggregations of *R. rhina* extended from the northern edge of Monterey Bay, and were situated at the outer continental shelf and upper slope, often in association with the headward parts of submarine canyon systems (Figure 3b). The center of *R. inornata* aggregation was located on the broad, inner continental shelf north of Monterey Bay and extended to the edge of the study region (Figure 4a), whereas that of *R. stellulata* was confined to a more limited region of the inner continental shelf to the south of Monterey Bay (Figure 4b). *Bathyraja kincaidii* biomass exhibited a single, highly significant hot spot cluster on the outer edge of the continental shelf from ∼37′ 10″ N–37′ 15″ N (Getis-G z-scores; Figure 4c).

**Figure 5:**
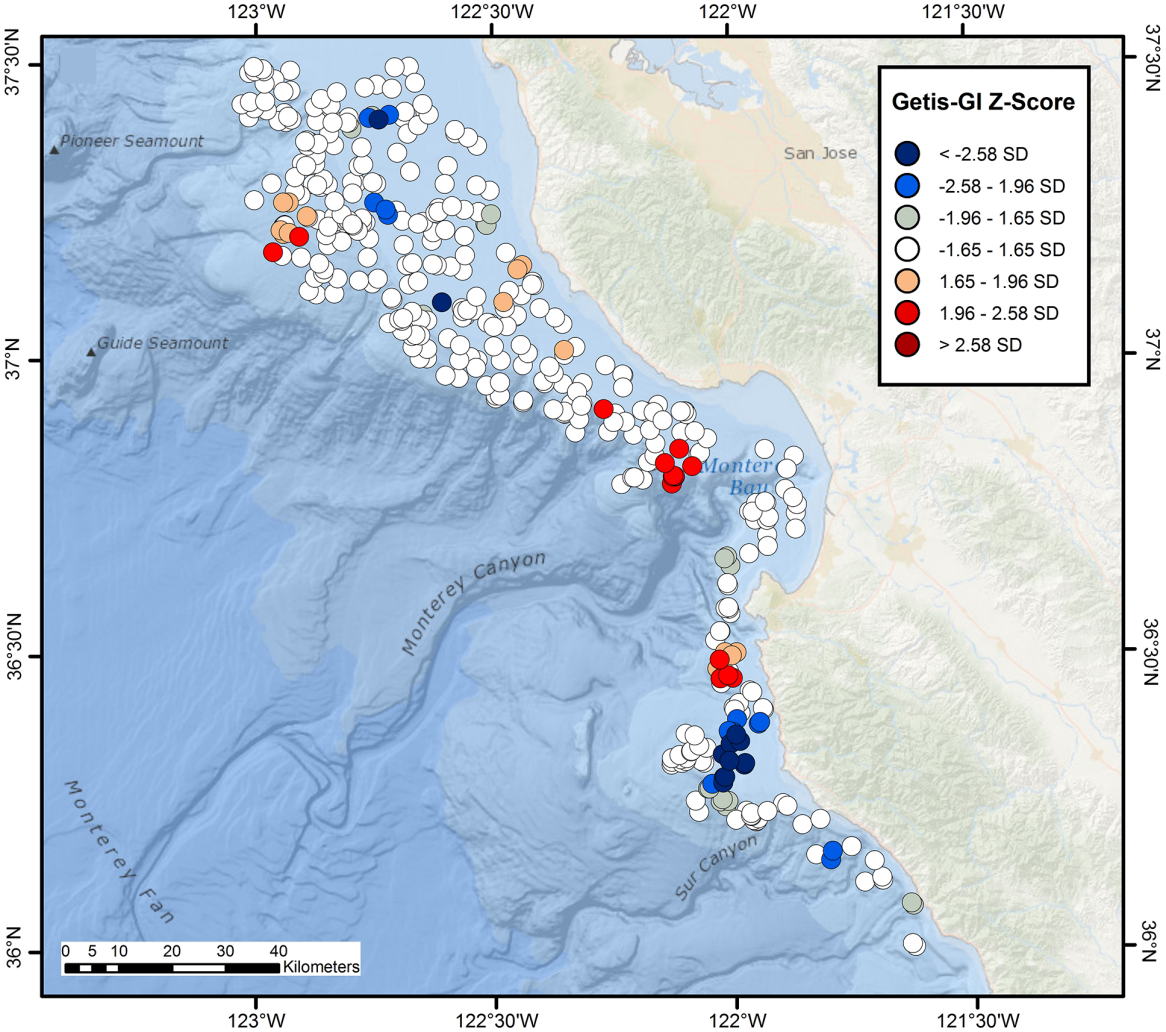
Hot and cold spot maps for species richness of all Californian skates. Getis-GI Hot spot Analysis Z-score plot of species richness of skates collected from NMFS–AFSC and NMFS–NWFSC trawl surveys conducted during 1997–2010.

Two skate species were typically caught in a tow (%FO = 52.8%, *n* = 124), but only 12.8% of tows (*n* = 30) contained three species, none contained four species, and a single haul caught all five (0.4%, *n* = 1) species. Observed and expected species richness values did not differ significantly, however, indicating that skates were not more clustered or segregated than expected among hauls (*χ* ^2^ = 4.62, *p* = 0.463, df = 4). The haul with all five species was observed at 114 m, to the south of the Monterey Bay Peninsula. Catches of three species were generally scattered throughout the continental shelf from the northern axis of Monterey Bay to the northern extent of the study site, and were most commonly composed of *R. rhina*, *B. binoculata*, and *R. inornata* co-occurrences. Significant hot spots for species richness were found on the outer continental shelf in the northern part of the study region, in outer Monterey Bay, and just south of the Monterey Peninsula (Figure 5). The largest and most significant cold spot for species richness was nearly adjacent to the southernmost hot spot cluster, and other spatially restricted cold spots were noted on the continental shelf off the northern and southern mainland coast (Figure 5).

#### Comparison of Size Distributions

When species commonly co-occurred in central California trawls, they consistently differed in size. Among 19 hauls that captured at least one *B. binoculata*, *R. rhina*, and *R. inornata*, mean TL differed significantly among species (*F* = 102.87, *p*<0.001). Mean TL of *B. binoculata* (88.3±28.6 cm, *n* = 33) in these hauls was significantly greater than that of *R. rhina* (51.9±19.0 cm, *n* = 92; Tukey’s HSD, p<0.001) and *R. inornata* (39.4±13.9 cm, *n* = 155; Tukey’s HSD, p<0.001), and specimens of *R. rhina* were significantly larger than those of *R. inornata* (Tukey’s HSD, p<0.001). When two species were caught in a haul, they were primarily composed of *R. rhina-B. kincaidii* (*n* = 69) and *R. rhina-R. inornata* (*n* = 45) co-occurrences. Mean size of *R. rhina* (47.8±17.5 cm TL, *n* = 232) was significantly greater than that of *R. inornata* (41.7±14.6 cm TL, *n* = 262; *t* = −4.20, *p*<0.001). Because the NMFS–NWFSC database did not contain length information for *B. kincaidii*, length data were instead culled from the more spatially restricted SWFSC–FED trawl data set for comparative purposes (Figure 1, Table 1). Among 53 hauls in Monterey Bay that captured both species, mean TL of *R. rhina* (56.4±16.4 cm, *n* = 912) was significantly greater than that of *B. kincaidii* (48.3±4.9 cm, *n* = 156; *t* = −6.16, *p*<0.001).

#### Regression Models

The central California skate assemblage exhibited depth zonation based on regression models constructed using NMFS–NWFSC data throughout the study range. Common distributional ranges and distribution of maximum catches were determined for *B. binoculata* and *B. kincaidii* on a species specific basis, and for juvenile and transitional/adult life stages of *R. rhina* and *R. inornata*. The distribution of *R. stellulata* was not analyzed because captures were extremely infrequent and of small magnitude. The common latitudinal ranges of all species spanned the study region. *Beringraja binoculata* and *R. inornata* juveniles and transitional/adult individuals typically were found from the inner continental shelf to the outer continental shelf or upper continental slope, respectively (Table 3). Common depth ranges and depth at maximum occurrence were similar between *R. inornata* life stages (Table 3). The distributions of *Raja rhina* and *B. kincaidii* were shifted to deeper water, with *B. kincaidii* occurring from the outer continental shelf to the maximum depth of the study region, and *R. rhina* extending from the mid-shelf to maximum depth (Table 3). The common depth range was equivalent between *R. rhina* life stages, but the maximum count of juveniles was found approximately 45 m shallower than that of adults (Table 3). Typical temperature ranges were warmer, and much more restricted for the two shallower-dwelling species (Table 3). *Raja rhina* was (numerically) the most abundant species among non-zero hauls, whereas *B. binoculata*, by contrast, was the least abundant (Table 3).

**Table 3:** ZIP model predictions of median and maximum count, and associated depth and latitude ranges, and GAM model predictions of temperature for central California skate species/life stages throughout the study site.

Model Outputs	*B. binoculata*	*R. rhina* (juv)	*R. rhina* (t-a)	*R. inornata* (j)	*R. inornata* (t-a)	*B. kincaidii*
Non-Zero Hauls (N)	30	149	138	97	66	80
Median Count	0.2	3.3	1.7	0.9	0.4	0.1
Common Depth (m)	58–182	124–598	124–598	58–231	58–242	173–598
Common Temp (C)	8.6–9.6	5.4–9.1	5.4–9.1	8.2–9.6	8.2–9.6	5.4–8.7
Common Lat	36.00–37.50	36.10–37.50	36.00–37.50	36.03–37.50	36.00–37.50	36.00–37.50
Max Count	1.3	12.3	6.0	8.2	3.3	18.1
Max Depth (m)	58	293	338	67	58	354
Max Latitude	36.00	36.80	36.74	37.13	37.01	36.92

N = number of hauls, m = meters, C = degrees Celcius, Max = maximum. Results were calculated from National Marine Fisheries Service–Northwest Fisheries Science Center trawl data collected during 2003–2010 among 235 tows throughout the study region. Common depth, temperature, and latitudinal range are included (see methods), as well as the depth and latitude at which the maximum number of skates were predicted.

Seasonal shifts in depth and abundance were evident in the central California skate assemblage from the greater Monterey Bay region. *Raja stellulata* catches were too low and infrequent in the SWFSC–FED trawl data set to enable modeling. The longline survey, however, exhibited much greater catch numbers and frequencies and was therefore used to estimate the distribution of this species on an overall basis (i.e., combined life stages). For all other skate species, predicted catches were greater for juveniles than transitional/adult specimens (Table 4). This trend was consistent for each species among all seasons, with the exception of *R. rhina*, and was most pronounced for *B. kincaidii*. Depth zonation was similar to that determined from the more expansive NMFS-NWFSC data set, with *B. binoculata* and *R. inornata* commonly found on the continental shelf during all seasons and *R. rhina* and *B. kincaidii* distributions extending to much deeper waters (Table 4). *Raja stellulata* typically occurred on the mid-shelf and its predicted seasonal depth of maximum counts ranged from 92–135 m. Juvenile *B. kincaidii* and, to a lesser extent, *B. binoculata* were found shallower than adults, whereas depth zonation between life stages was not evident for *R. rhina* or *R. inornata* (Table 4). Median and maximum predicted counts of juvenile *B. binoculata* and *B. kincaidii*, transitional and adult male *R. inornata*, and both life stages of *R. rhina* were most abundant during the Oceanic season. The common depth range of *B. binoculata* juveniles and transitional/adults deepened throughout the year (Davidson < Oceanic < Upwelling), whereas juvenile and transitional/adult male *R. inornata* exhibited the opposite trend (Table 4). No seasonal differences in depth zonation were evident for life stages of the deeper dwelling *R. rhina* and *B. kincaidii* (Table 4). Sample sizes were low for all species and life stages. Therefore, though these results from Monterey Bay are useful for comparative purposes with the more expansive NMFS–NWFSC data set, they should be considered preliminary.

**Table 4:** ZIP model predictions of median and maximum count, and associated depth ranges, and GAM model predictions of temperature for life stages of central California skates in Monterey Bay.

Species	Life Stage	Season	Catches	Hauls	Median Count	Common Depth	Common Temp	Pred. Max Count	Pred. Depth	Pred. Temp
				N		m	C		m	C
*B. binoculata*	J	Davidson	22	46	6.3	14–85	9.4–9.9	7.9	18	9.9
*B. binoculata*	J	Oceanic	22	46	13.7	14–113	9.2–9.9	21.2	35	9.8
*B. binoculata*	J	Upwelling	22	46	6.3	38–164	8.8–9.7	9.2	79	9.4
*B. binoculata*	TR, A	Davidson	14	46	1.5	21–152	8.9–9.9	2.8	59	9.5
*B. binoculata*	TR, A	Oceanic	14	46	0.9	30–164	8.8–9.8	1.5	68	9.5
*B. binoculata*	TR, A	Upwelling	14	46	0.8	63–318	7.6–9.5	1.3	141	9.3
*R. rhina*	J	Davidson	28	35	23.0	113–342	7.4–9.2	40.3	204	8.5
*R. rhina*	J	Oceanic	28	35	33.6	32–532	5.9–9.8	54.1	426	6.7
*R. rhina*	J	Upwelling	28	35	10.4	91–368	7.2–9.3	13.7	176	8.7
*R. rhina*	TR, A	Davidson	24	35	9.6	91–459	6.5–9.3	11.2	204	8.5
*R. rhina*	TR, A	Oceanic	24	35	28.3	98–532	5.9–9.3	37.7	396	7.0
*R. rhina*	TR, A	Upwelling	24	35	13.8	73–532	5.9–9.5	17.6	204	8.5
*R. inornata*	J	Davidson	21	46	5.9	24–318	7.6–9.8	8.5	98	9.3
*R. inornata*	J	Oceanic	21	46	5.3	44–190	8.6–9.7	7.1	98	9.3
*R. inornata*	J	Upwelling	21	46	15.7	32–113	9.2–9.8	28.8	59	9.6
*R. inornata*	TR, AM	ALL	18	46	1.4	30–176	8.7–9.8	1.7	91	9.3
*R. inornata*	TR, AM	Davidson	18	46	1.3	26–220	8.3–9.8	1.8	79	9.4
*R. inornata*	TR, AM	Oceanic	18	46	8.8	30–176	8.7–9.8	20.1	73	9.5
*R. inornata*	TR, AM	Upwelling	18	46	3.7	35–141	8.9–9.8	7.1	68	9.5
*R. stellulata*	ALL	Davidson	24	106	1.8	84–123	9.1–9.4	5.9	102	9.3
*R. stellulata*	ALL	Oceanic	24	106	0.9	70–260	8.0–9.5	1.9	135	9.0
*R. stellulata*	ALL	Upwelling	24	106	0.9	70–112	9.2–9.5	1.9	92	9.2
*B. kincaidii*	J	Davidson	13	58	23.0	113–342	7.4–9.2	40.3	204	8.5
*B. kincaidii*	J	Oceanic	13	58	33.6	32–532	5.9–9.8	54.1	426	6.7
*B. kincaidii*	J	Upwelling	13	58	10.4	91–368	7.2–9.3	13.7	176	8.7
*B. kincaidii*	TR, AF	ALL	22	58	1.8	78–614	5.3–9.4	3.5	309	7.6
*B. kincaidii*	TR, AM	Davidson	22	58	2.9	143–614	5.3–8.9	4.2	400	6.9
*B. kincaidii*	TR, AM	Oceanic	22	58	1.2	47–614	5.3–9.7	10.8	614	5.3
*B. kincaidii*	TR, AM	Upwelling	22	58	1.1	72–614	5.3–9.5	2.6	614	5.3

J = juvenile, TR = transitional, A = adult, M = male, F = female, ALL = combined life stages, N = number of hauls, m = meters, C = Celsius. Results were calculated from NMFS–SWFSC–FED trawl and longline data (*R. stellulata*, only) collected during 2002–2005 in the greater Monterey Bay region. Oceanographic season, catches with skates (Catches) and the total number of trawl or longline catches (Hauls) are included, as well as common depth and temperature ranges, and depth and temperature at which the maximum number of occurrences was predicted. Predicted median count was used as the cut-off value to determine common ranges.

#### Habitat Association Analysis

Skates were infrequently observed during manned submersible operations off central California, with only 190 occurrences documented among 1203 dive transects totaling 0.593 km^2^ of seafloor. Transect habitat composition was as follows: Hard (0.190 km^2^, *n* = 389), Mixed (0.298 km^2^, *n* = 618), and Soft (0.105 km^2^, *n* = 196). *Raja rhina* was the most numerically abundant species among transects, and was significantly more abundant on mixed and soft habitat than on hard habitat (Chi-square; Table 5). Based on ZIP model results, occurrence was significantly greater on mixed sediment submersible transects as compared to soft habitat transects (*z* = 1.99, *p* = 0.046), and latitude at the maximum predicted count (36.69° N) was consistent with ZIP model results calculated from NMFS–NWFSC trawl survey data (Table 3). The depth associated with the predicted maximum count (221 m) was considerably shallower when calculated using submersible data, as a consequence of the much shallower distribution of submersible transects compared to NMFS–NWFSC bottom trawls (Table 1). Although far less commonly observed, *R. stellulata* also was encountered significantly more often on soft and mixed substrate transects (Chi-square; Table 5). *Raja inornata* and *B. kincaidii* displayed strong associations with soft substrate, whereas the few *B. binoculata* observed were all on transects of predominantly mixed substrates. All skate species displayed substantial variation in coloration, which they appeared to modify to match the surrounding seafloor. This variability was most evident in *R. rhina*, *B. binoculata*, and *R. stellulata*, all of which were more commonly observed on heterogeneous seafloors, and could exhibit black or white spotting, mottling, or markings or be of uniform coloration (browns to greys), depending on their surroundings.

**Table 5:** Descriptive statistics, depth range, and mesoscale habitat electivity for central California skates as calculated from manned submersible data in the greater Monterey Bay region.

Species	*n*	%FO	Density	Depth Range	Hard	Mixed	Soft
			#/km^2^	m			
*Beringraja binoculata*	4	0.33	6.8	89–275	0.00	1.00*	0.00
*Raja rhina*	137	7.73	232.2	79–313	0.15	0.43*	0.42*
*Raja inornata*	5	0.42	8.5	91–290	0.00	0.15	0.85*
*Raja stellulata*	16	1.33	27.1	58–138	0.18	0.37*	0.45*
*Bathyraja kincaidii*	10	0.83	16.9	79–301	0.11	0.09	0.80*

*n* = sample size, %FO = frequency of occurrence, km = kilometers, m = meters.

* = p<0.05 for Chi-square test. Number (*n*), frequency of occurrence (%FO), density (#/km^2^), depth range and mesoscale habitat electivity (after Manly et al. 1993) of the California skate assemblage among 1203 submersible dive transects conducted at depths of 24–316 m.

Of 1002 skates observed in 3775.1 hours of bottom time during ROV dives, 977 were identified to species, including: 771 *R. rhina*, 197 *B. kincaidii*, 6 *B. binoculata*, 1 *R. stellulata*, 1 roughtail skate (*B. trachura*), and 1 deepsea skate (*B. abbysicola*). With the notable exception of *R. rhina*, hardnose skates were largely absent from this data set. *Beringraja binoculata* individuals ranged from 30–243 m and were observed resting on flat, sand- (*n* = 4) or mud-covered (*n* = 1) seafloors or swimming demersally (*n* = 1), typically within larger regions of uniform habitat type. One *R. stellulata* was observed at 103 m on a flat, lower portion of a heavily sedimented rock wall. The maximum depths recorded for the deeper dwelling species among all ROV dives performed by MBARI throughout the West Coast are: *R. rhina* (931 m), and *B. kincaidii* (786 m). There were no verified sightings of *R. inornata*.

Off central California, *R*. *rhina* was observed at depths of 74–599 m, with a median depth of 340 m (Q_1_ = 279 m, Q_3_ = 388 m). Most (94.6%, *n* = 686) individuals were found on mud, but a small percentage was found on rock, including pebble, cobble, scarps and outcrops in particular. Two specimens were observed swimming demersally. Most associated microhabitats were of low-relief (91.0%). Macrohabitat types were of greater relief (medium = 13.2%, high = 20.4%) and contained a higher percentage of rock (26.6%) than microhabitats. No obvious sexual segregation was observed. Instead, subadult and adult males and females and juveniles were found throughout the canyon system, especially on the upper slope, and were frequently encountered at the shelf break. Median depth of juveniles (276 m, *n* = 196) was substantially shallower than that of subadults and adults (361 m, *n* = 255). *Raja rhina* commonly employs its walking legs (anterior lobes of pelvic fins) for locomotion, especially at short distances, and exhibited considerable dexterity. One individual was observed performing traditional punting for fairly rapid, small-scale movements, with one specimen using this technique to hurdle a detached giant kelp (*Macrocystis pyrifera*) holdfast. *Raja rhina* also used its walking legs individually to pivot in place, or alternately to slowly walk across the sediment. It was observed fluidizing sediment to enable partial burying for resting and/or refuge.

*Bathyraja kincaidii* was found from depths of 46–596 m, with a median depth of 400 m (Q_1_ = 320 m, Q_3_ = 455 m). Individuals were typically found on mud (92.9%, *n* = 183), but a small percentage was associated with rock, including cobble, boulder fields, scarps and especially outcrops. Correspondingly, most associated microhabitats were of low relief (91.5%). Macrohabitat types displayed a greater association with medium- (8.5%) and high-relief (12.8%) seafloors, and contained a higher percentage of rock (17.5%), but were generally consistent with microhabitat findings. No obvious sexual segregation was observed. An aggregation of juveniles (*n* = 20) was noted between 451–564 m on a single dive conducted during March 2007 on the central portion of the northern canyon system. *Bathyraja kincaidii* was occasionally observed using its walking legs to punt; however, its primary form of locomotion is undulatory swimming with an exaggerated, flapping motion. *Bathyraja kincaidii* often skims the benthos as it swims, burst swimming and relocating quickly when startled, as indicated by a trailing plume of sediment. Like *R. rhina*, *B. kincaidii* was observed fluidizing sediment to enable partial burying.

**Figure 6:**
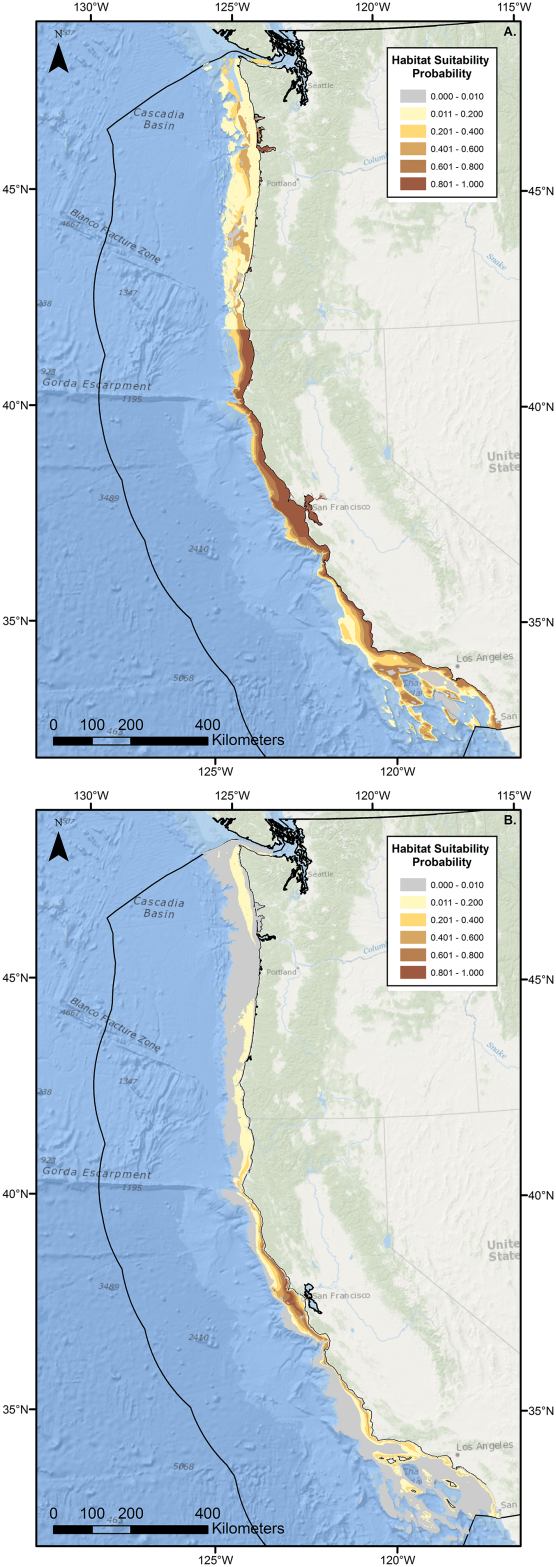
Original and updated habitat suitability probability profiles for *Raja inornata*. Reconstruction of original (A) and updated (B) habitat suitability probability profiles for adult and transitional/adult *Raja inornata* throughout the U.S. West Coast. The original profile (A) was depicted in the 2005 Essential Fish Habitat Amendment to the Pacific Coast Groundfish Fishery Management Plan (Anonymous 2005). The black line offshore depicts the limit of the U.S. EEZ.

The HSP profile of *R. inornata* adults was substantially modified based on the results of this study (Figure 6a). Sizes were generally consistent between adult designations of *R. inornata* as defined by NMFS from anecdotal data (>52 cm TL), and transitional/adult designations as defined in this study from unpublished size-at-maturity information (males >50.1 cm TL, females >54.5 cm TL). All of the general input parameters (latitude, depth, benthic habitat) were updated, resulting in a new HSP map that bears little resemblance to the original (Figure 6b). The most notable difference between the two profiles is the significant reduction in suitable habitat in the updated profile (Figure 6). Maximum depth for this species was reduced from 1600 m to 318 m, with the prior depth record consisting of a misidentification. This correction moved the distribution of this species considerably inshore (Figure 6). In addition, highly suitable habitat (HSP >0.80) was greatly reduced and restricted to a region of central California off San Francisco Bay (Figure 6b). Habitat suitability also showed a much more pronounced latitudinal gradient in the updated map, with all HSP values >0.2 contained entirely within California and poor habitat quality located off the Oregon and Washington coasts (Figure 6b).

### Western Gulf of Alaska Skate Assemblage

#### Spatial Analysis

Clustering of skate distributions in the western Gulf of Alaska was highly significant at the global scale, driven by the greater intensity of highly clustered high catch rates over lows. Global spatial autocorrelation was marked for both measures of all species (Global Moran’s I; Table 6). Z-scores for all General G statistic calculations were positive, indicating that spatial highs were of significantly greater influence than spatial lows for this data set. When individuals of all species were considered, the results mirrored those of single species (Table 6).

**Table 6:** Spatial analysis results for the western Gulf of Alaska skate assemblage.

Species	N	WT	%FO	Measure	ISA	Global Moran's I	K	General G
					km		km	
*Beringraja binoculata*	883	14,195	10.6	CPUE (N)	40.36*	0.083**	20.06*	0.048**
				CPUE (WT)	40.36*	0.091**	24.85*	0.057**
*Raja rhina*	1340	9128	21.6	CPUE (N)	NS	0.127**	26.87*	0.011**
				CPUE (WT)	NS	0.090**	27.47*	0.011**
*Bathyraja aleutica*	768	5961	11.1	CPUE (N)	76.52*	0.072**	29.82*	0.124**
				CPUE (WT)	37.51*	0.098**	29.48*	0.047**
*Bathyraja interrupta*	720	1386	12.1	CPUE (N)	58.44*	0.081**	29.57*	0.070**
				CPUE (WT)	34.34*	0.069**	30.30*	0.033**
*Bathyraja parmifera*	128	541	2.7	CPUE (N)	46.39*	0.020**	36.83*	0.064**
				CPUE (WT)	37.35*	0.021**	41.87*	0.050**
All Species	3839	31,211	43.0	Species Richness	NS	0.209**	24.26*	0.009**

NS = not significant. N = number, WT = weight (kilograms), %FO = frequency of occurrence among hauls, CPUE = catch-per-unit-effort (#/km^2^, kg/km^2^), ISA = incremental spatial autocorrelation, K = K Function, km = kilometers. Significant p-value thresholds are Indicated as follows: * = <0.05, ** = <0.001. Data were collected during National Marine Fisheries Service, Alaska Fisheries Science Center trawl surveys (n = 3931) conducted during 1999–2011 on the continental shelf and slope of the western Gulf of Alaska.

Local clustering was significant at peak intervals and over broad spatial scales for four skate species in the western GOA. ISA yielded significant results by both measures for all species but *R. rhina* (Table 6). Clustering exhibited similar significant maximum peaks among species by weight, at distances ranging from 34.34–40.36 km. Numerical CPUE results of ISA were more variable and typically occurred at greater distances (Table 6). Clustering was highly significant at scales ranging from 20.06 km (*B. binoculata*, number) to 41.87 km (*B. parmifera*, biomass) but was quite similar between measures for all species (K; Table 6). In each case, cluster distance was comparable or slightly larger by weight (K; Table 6). When individuals of all species were combined, there were no significant peak clusters. Skates distribution was significantly more clustered than random, however, at distances of up to 24.26 km.

**Figure 7:**
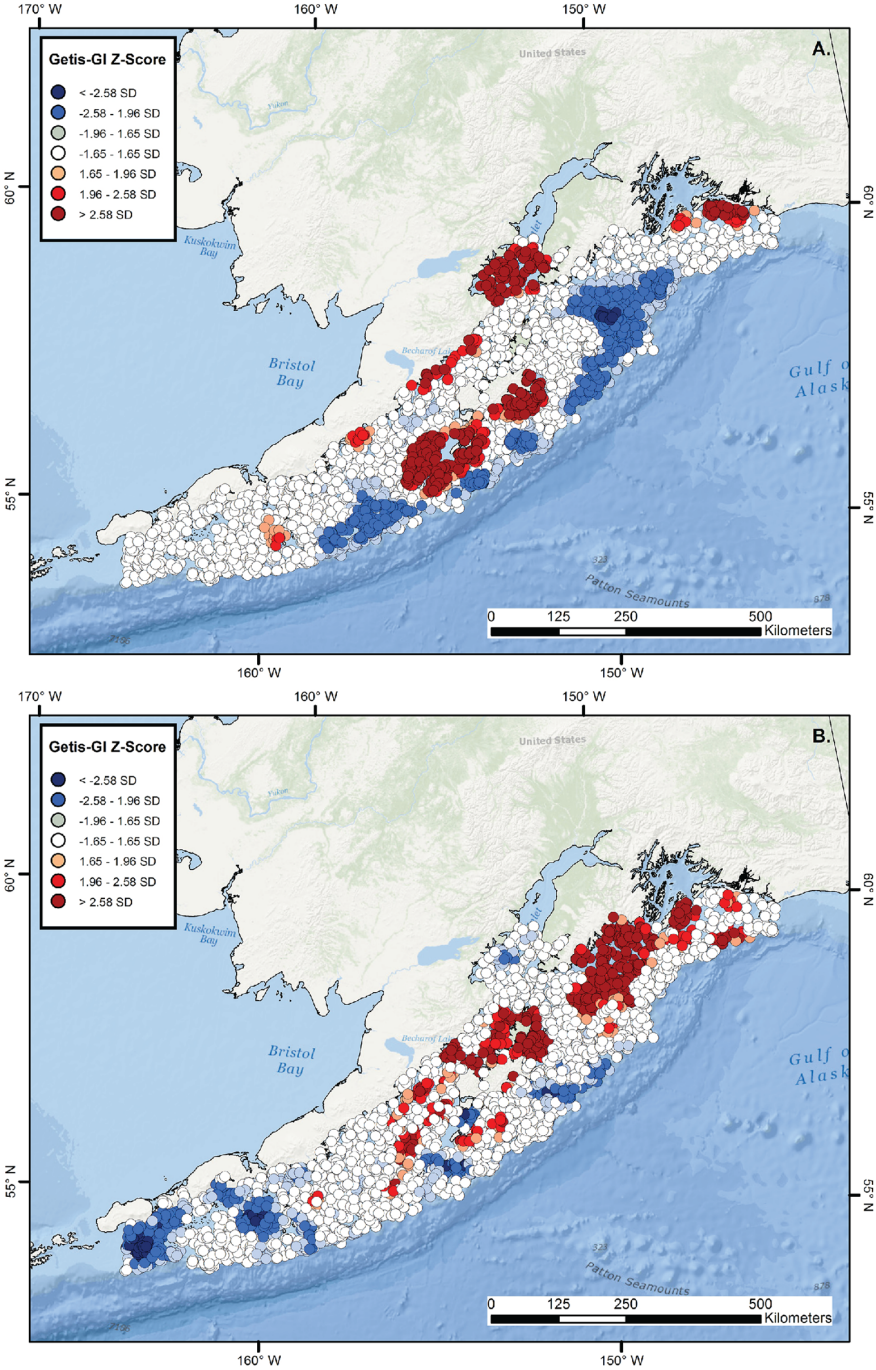
Hot and cold spot maps for *Beringraja binoculata* and *Raja rhina*. Getis-GI Hot Spot Analysis Z-score plots of catch-per-unit-effort (kg/km) for the hardnose skates, *Beringraja binoculata* (A) and *Raja rhina* (B), in the western Gulf of Alaska, as calculated from NMFS–AFSC trawl surveys conducted during 1999–2011.

**Figure 8:**
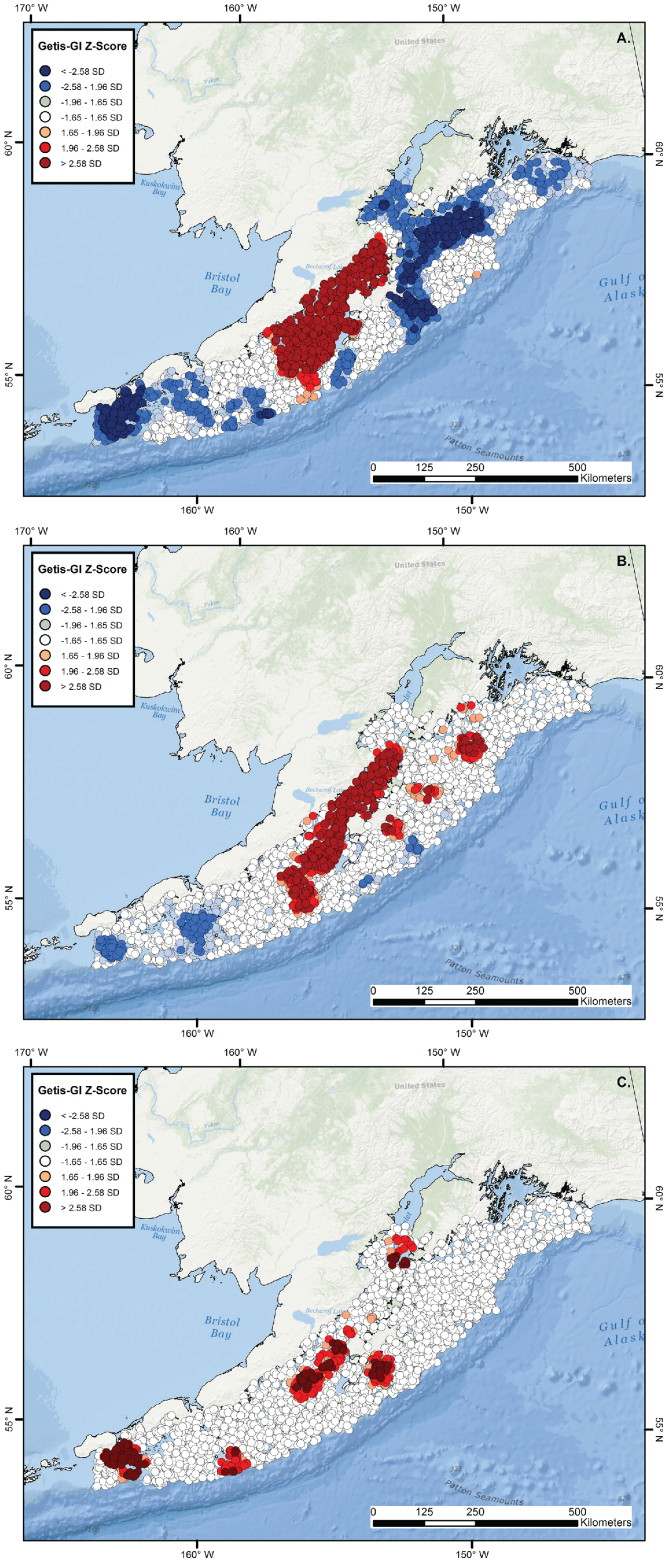
Hot and cold spot maps for *Bathyraja aleutica, B. interrupta,* and *B. parmifera*. Getis-GI Hot Spot Analysis Z-score plots of catch-per-unit-effort (kg/km) for the softnose skates, *Bathyraja aleutica* (A), *B. interrupta* (B), and *B. parmifera* (C), in the western Gulf of Alaska, as calculated from NMFS–AFSC trawl surveys conducted during 1999–2011.

Significant hot and cold spots were observed for skates in the western GOA, with several clusters and/or expansive, highly clustered areas located within the study region. Significant aggregations of *B. binoculata* and *R. rhina* were spatially segregated. Highly significant, long-term regions of *B. binoculata* aggregation were located along the mainland coast (especially lower Cook Inlet), on the continental shelf southwest of Kodiak Island, and off the Island’s east coast (Figure 7a). By contrast, cold spots were less significant and located in regions of relatively deep water at the outer edge of the survey region and just south of the Kenai Peninsula (Figure 7a). The same general region south of the Kenai Peninsula contained the greatest concentration of clustered *R. rhina* biomass values, with a smaller, more diffuse region extending from northeastern Shelikof Strait to the offshore waters north of Kodiak Island (Figure 7b). Additional, localized hot spots were scattered throughout the eastern and central portion of the study region, whereas the greatest concentration and most highly significant low values occurred at the western extent (Figure 7b). The *B. aleutica* population was highly significantly concentrated from the greater Shelikof Strait region to the continental shelf region southwest of Kodiak (Figure 8a). A similarly expansive region of clustered low biomass values extended from southern Cook Inlet directly offshore and from the southern coast of the Kenai Peninsula to deeper, offshore regions (Figure 8a). The primary region of *B. interrupta* aggregation also was located throughout Shelikof Strait (Figure 8b). Spatially restricted, positive clustering was observed on the outer shelf in the east-central portion of the study region, and similar, isolated cold spots were located in the central and especially western part of the study site (Figure 8b). Highly significant hot spots were observed for *B. parmifera* at the western extent of the study region, with more spatially restricted, less significant clustering occurring on the continental shelf south and east of Kodiak, in southern Cook Inlet, and at the outer edge of the continental shelf at ∼158°W–159°W (Figure 8c).

The great majority of tows contained no skates (57.0%, *n* = 2241) or one species (30.6%, *n* = 1202). Tows with multiple species were infrequently observed. Only ten tows (0.3%) contained four species and no tows contained all five species. Observed and expected values differed significantly, with tows of zero, four, and five species occurring less often than expected by chance and tows with one species occurring more often (*χ* ^2^ = 65.40, *p*<0.001, df = 4). Tows with multiple species mainly were located in the central and north-eastern part of the study region, with the region of greatest species richness occurring in the greater Shelikof Strait region and waters south and west of Kodiak Island. In all cases, hauls with four species did not contain *B. parmifera*.

**Figure 9:**
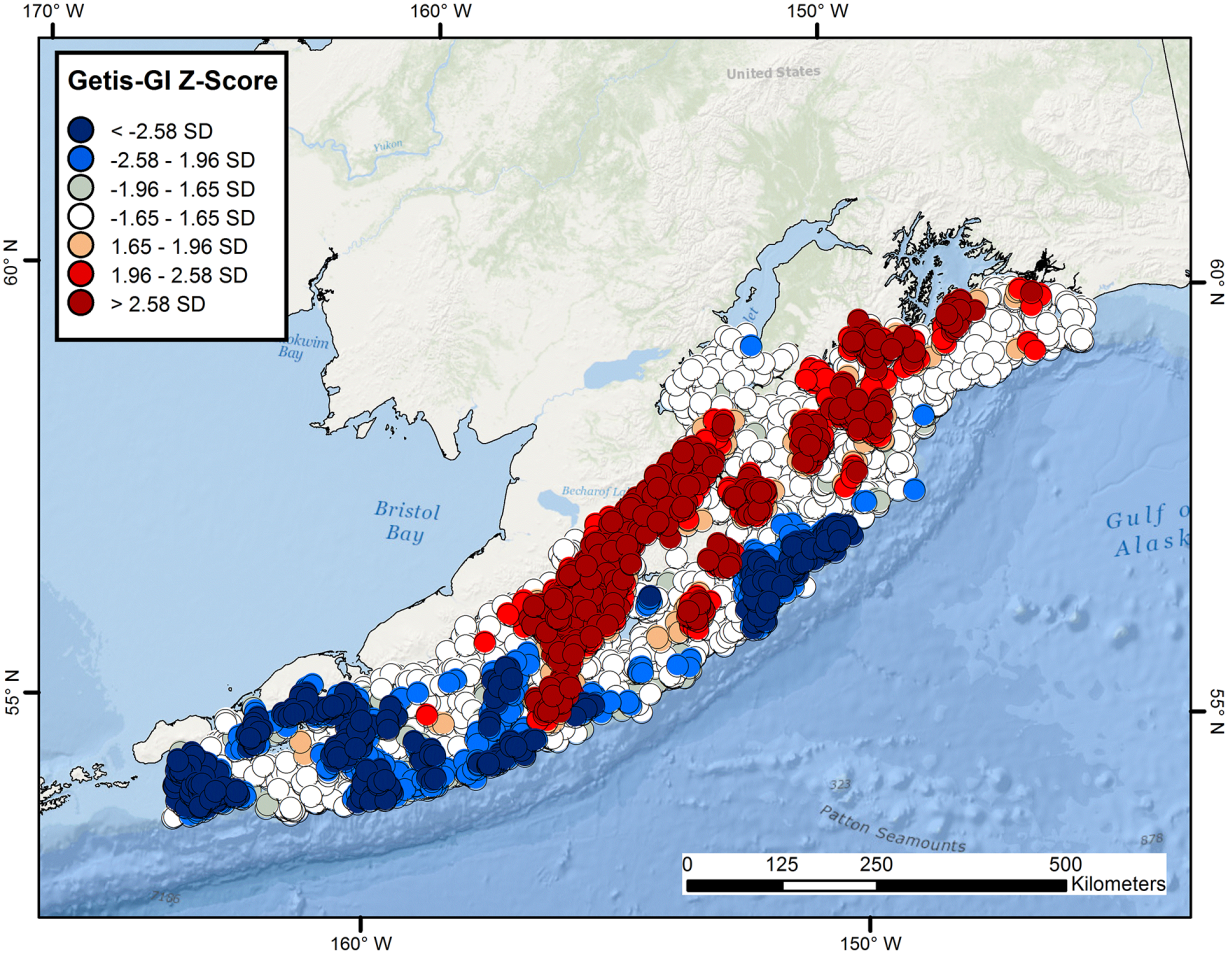
Hot and cold spot maps for species richness of Alaskan skates. Getis-GI Hot Spot Analysis Z-score plot of species richness of skates collected during combined NMFS–AFSC trawl surveys conducted in the western Gulf of Alaska during 1999–2011.

Highly significant clustering of highs and lows was evident throughout the study region for combined skates of all species. An expansive, contiguous cluster of highly significant positive values for species richness was located in the greater Shelikof Strait region, with more restricted regions evident throughout the central and north-eastern portion of the study site (Fig. 9). Cold spots were largely clustered in the western and outermost central portion of the region (Figure 9).

#### Comparison of Size Distributions

When species commonly co-occurred in trawls conducted in the western Gulf of Alaska, they typically differed in size. Among the 10 hauls that captured four species, mean TL differed significantly among species (Table 7). Mean TL of *B. binoculata* was significantly greater than that of all other species, whereas *B. interrupta* was significantly smaller (Table 7). No difference was noted between mean TL of *R. rhina* and *B. aleutica*, but the power to detect a difference was extremely low (Table 7, Power = 0.04). The majority (60%) of tows with three species (*n* = 55) caught *R. rhina*, *B. aleutica*, and *B. interrupta*. Mean TL of these species differed significantly, as did all post-hoc comparisons (Table 7). Among co-occurring species pairs, mean size of (larger indicated first) *R. rhina*–*B. interrupta* (*n* = 141 tows), *B. aleutica*–*B. interrupta* (*n* = 44 tows), and *B. binoculata*–*B. interrupta* (*n* = 24 tows) differed significantly (Table 7). *Raja rhina*–*B. binoculata* (*n* = 42 tows) and *R. rhina*–*B. aleutica* (*n* = 82 tows) mean TL did not differ significantly (Table 7). Relatively high standard deviations resulted in low power for these comparisons (Power = 0.13, 0.05; respectively).

**Table 7:** ANOVA and t-test comparisons of mean total length among co-occurring skates in the western Gulf of Alaska.

Species Comparison	*n*	TL (cm)	SD	F/t-Statistic	p-value
*B. binoculata*	10	136.9	22.3	18.54	<0.001
*B. interrupta*	17	67.5	10.0		
*R. rhina*	19	108.7	25.0		
*B. aleutica*	17	107.2	34.0		
*B. interrupta*	96	65.6	14.3	83.87	<0.001
*R. rhina*	117	96.6	26.3		
*B. aleutica*	114	106.1	25.4		
*R. rhina*	101	105.4	23.9	0.818	0.414
*B. aleutica*	143	108.2	27.8		
*B. interrupta*	69	65.9	14.5	14.373	<0.001
*B. aleutica*	95	111.2	25.5		
*B. interrupta*	193	66.0	12.3	–19.579	<0.001
*R. rhina*	252	104.4	25.0		
*B. binoculata*	61	110.4	40.5	0.294	0.769
*R. rhina*	69	108.8	20.3		
*B. binoculata*	54	108.9	37.0	–5.308	<0.001
*B. interrupta*	29	71.3	12.0		

*n* = number of individuals, TL (cm) = mean total length, SD = standard deviation. Comparisons are based on co-occurrence among NMFS-AFSC trawls conducted in the western Gulf of Alaska during 1999–2011.

#### Regression Models

The western GOA skate assemblage exhibited depth and spatial zonation based on results of regression models constructed using NMFS–AFSC data throughout the study range. Skates in the GOA were far less frequently encountered and exhibited much lower catch rates than those off central California (Table 2, Table 6). Individuals of all life stages of *B. binoculata* exhibited very similar common depth and longitudinal ranges, and depths of maximum occurrence (Table 8). Distribution of *R. rhina* life stages was variable. Transitional specimens had the widest (and deepest) typical depth range, and the distribution of adults was shifted to more shallow waters than that of juveniles (Table 8). Depth of maximum occurrence, however, decreased with ontogeny, and juveniles commonly occurred over a much broader longitudinal range than transitionals or adults (Table 8). The typical temperature range for *R. rhina* was more restricted and slightly lower in the GOA than off central California (Table 3, Table 8). *Bathyraja aleutica* exhibited the same trend in maximum occurrence as *R. rhina*, with juveniles found at deepest depths and adults at shallowest. The depth ranges of maximum occurrence, as well as the common depth ranges, were shifted to deeper water for life stages of this species, and the center of distribution occurred more westward than that of *R. rhina* (Table 8). Correspondingly, *B. aleutica* commonly occurred across a temperature range that was slightly warmer than that of *R. rhina* (Table 8). Depth and spatial zonation differed somewhat between life stages of *B. interrupta*, but differences were not of substantial magnitude or consistent across ontogeny (Table 8). The depth range of this species overlapped considerably with that of *R. rhina* (slightly shallower) and *B. aleutica* (slightly deeper), but the longitudinal range of common occurrence was shifted west of *R. rhina*, and the longitude at maximum occurrence was distinct (Table 8). *Bathyraja parmifera* was captured infrequently and in very low numbers, but was encountered over the widest depth range of all species (Table 8). Transitional individuals were commonly found both shallower and substantially deeper than juveniles, at temperatures ranging from (2.7°C–8.0°C). However, results should be considered preliminary for this species because of extremely low and infrequent counts. The maximum predicted occurrence of this species was at the western extent of the study region. For all species, median predicted occurrences of juveniles were greater than that of older life stages, and maximum predicted occurrences were always greatest for juveniles (Table 8).

**Table 8:** Logistic model predictions of median and maximum count, and associated depth and longitude ranges, and GAM model predictions of temperature for life stages of skates in the western Gulf of Alaska.

Species	LifeStage	Catches	Hauls	MedianO.P.	CommonDepth	CommonTemp	CommonLongitude	Max.O.P.	Depth	Temp	Pred.Long
			N		m	C	W		m	C	W
*B. binoculata*	J	208	3908	0.10	13–91	5.4–8.0	144.01–164.00	0.87	13	8.0	–144.01
*B. binoculata*	TRF	26	3908	0.10	13–58	6.2–8.0	144.01–159.92	0.86	13	8.0	–144.01
*B. binoculata*	TRM	157	3908	0.05	13–83	5.5–8.0	144.01–164.00	0.14	29	7.4	–164.00
*B. binoculata*	A	149	3908	0.05	13–91	5.4–8.0	144.01–163.18	0.22	22	7.6	–152.57
*R. rhina*	J	306	3894	0.20	129–373	4.5–5.1	144.01–151.35	0.35	219	5.2	–144.01
*R. rhina*	TR	479	3894	0.10	58–407	4.4–6.2	144.01–157.88	0.26	154	5.2	–148.49
*R. rhina*	A	218	3894	0.05	58–286	4.7–6.2	145.23–157.47	0.12	129	5.1	–151.35
*B. aleutica*	J	261	3900	0.10	141–755	3.5–5.1	148.90–164.00	0.27	341	4.6	–157.47
*B. aleutica*	TR	159	3900	0.05	99–486	3.9–5.2	149.72–160.33	0.12	219	5.2	–155.02
*B. aleutica*	A	85	3900	0.02	91–373	4.5–5.4	149.31–161.14	0.07	184	5.3	–155.43
*B. interrupta*	J	168	3905	0.05	129–444	4.1–5.4	146.05–159.92	0.16	240	5.0	–152.98
*B. interrupta*	TRF	199	3905	0.05	108–373	4.5–5.1	145.23–160.33	0.15	201	5.3	–152.57
*B. interrupta*	TRM	173	3905	0.05	91–530	4.0–5.4	148.09–157.88	0.11	219	5.2	–152.98
*B. parmifera*	J	45	3922	0.02	91–407	4.4–5.4	155.84–164.00	0.04	184	5.3	–164.00
*B. parmifera*	TR	42	3922	0.02	13–984	2.7–8.0	162.37–164.00	0.02	13	8.0	–164.00

O.P. = Occurrence probability, J = juvenile, TR = transitional, F = female, M = male, A = adult, N = number of hauls, m = meters, C = degrees Celsius, W = West. Data were collected during 1999–2011 throughout the study region. Catches with skates (Catches) and the total number of number of trawl hauls (Hauls) are included, as well as common depth, temperature, and longitude ranges, and depth, temperature, and longitude at which the maximum number of occurrences was predicted.

Predicted depths of occurrence and magnitude of catches varied considerably among skate species and ADFG management regions. The largest predicted counts of *B. binoculata* were in Shelikof Strait, with lowest values associated with the Alaska Peninsula region. Predicted depth at maximum occurrence was consistent among regions and occurred in shallow shelf waters (Table 9). Predicted common depths, however, varied considerably among regions at depths ≤155 m. *Raja rhina* occurrence was greatest at Kodiak and Shelikof, with the maximum count (3.92/tow) predicted at 176 m off Kodiak Island. When compared with *B. binoculata* distribution, greater counts of *R. rhina* were evident at Kodiak and Kamishak, with greater median but smaller maximum values at Shelikof, and fewer *R. rhina* off the Alaska Peninsula (Table 9). Depths of common *R. rhina* occurrence extended from the mid**-**shelf to the outer shelf or upper slope. Like *B. binoculata*, predicted maximum depths of *R. rhina* occurrence were consistent among regions (176–178 m), but predicted common depth ranges were highly variable (Table 9). *Bathyraja interrupta* was relatively common among all regions, with median and maximum predicted catches exceeding those of *B. binoculata* and *R. rhina* in each management region. Depth ranges were similar to those of *R. rhina* in each region but Shelikof, where *B. interrupta* extended into considerably deeper waters (Table 9). Depths of maximum predicted catches, however, were considerably deeper than those of *R. rhina* at all locations except Kamishak (Table 9).

**Table 9:** ZIP model predictions of median and maximum count, and associated depths for skates among surveyed regions in the western Gulf of Alaska.

Species	Region	Catches	Hauls	Median	Common Depth	Max Count	Max Depth
					m		m
*B. binoculata*	Kamishak	76	160	1.39	33–78	1.44	51
*B. binoculata*	Kodiak	608	1834	1.50	22–121	1.94	49
*B. binoculata*	Peninsula	354	1363	0.86	26–101	1.11	51
*B. binoculata*	Shelikof	109	277	1.94	59–155	4.13	59
*R. rhina*	Kamishak	68	159	2.33	68–178	2.55	178
*R. rhina*	Kodiak	1242	1834	3.71	121–252	3.92	176
*R. rhina*	Peninsula	218	1363	0.45	102–210	0.5	176
*R. rhina*	Shelikof	225	277	3.57	146–208	3.61	177
*B. aleutica*	Kodiak	453	1834	0.81	132–252	2.52	252
*B. aleutica*	Peninsula	229	1363	0.57	106–210	1.75	210
*B. aleutica*	Shelikof	127	277	3.52	155–289	8.44	289
*B. interrupta*	Kamishak	95	153	5.66	66–178	6.32	178
*B. interrupta*	Kodiak	756	1834	3.05	121–252	3.81	252
*B. interrupta*	Peninsula	612	1363	3.62	101–210	4.32	210
*B. interrupta*	Shelikof	149	277	4.54	155–289	5.57	289
*B. parmifera*	Kodiak	301	1834	0.57	124–219	0.61	165
*B. parmifera*	Peninsula	160	1363	0.36	104–210	0.42	166
*B. parmifera*	Shelikof	42	277	0.20	139–199	0.2	166

m = meters. Data were collected from ADFG trawl surveys conducted in the west-central portion of the study region during 2003–2012. Sampling areas (Region), catches with skates (Catches) and the total number of trawls (Hauls) are included as well as common depth ranges, and depth at which the maximum number of occurrences was predicted. Median count was used as the cut-off value to predict common ranges.

*Bathyraja aleutica* and *B. parmifera* were rare in Kamishak surveys and their distributions were therefore only modeled for three management regions. *Bathyraja aleutica* was relatively abundant in Shelikof Strait, where it had the greatest maximum predicted catch of any species (Table 9). The common depth range of *B. aleutica* overlapped substantially with that of *R. rhina* and *B. interrupta*, whereas the maximum predicted depths mirrored that of *B. interrupta*. As previously indicated, however, mean sizes of these specimens differed significantly. *Bathyraja parmifera* occurrence was infrequent among all management regions, but common and maximum predicted catches were greatest at Kodiak and least at Shelikof (Table 9). This species was primarily distributed on the outer continental shelf with only slight variability among regions (Table 9).

## Discussion

Spatial segregation among ENP skates was pronounced (Q2), and supports a general conclusion that comparably sized skate individuals of different species do not typically co-occur (Q3). In both study regions, species distributions exhibited temporally consistent areas of high aggregation that were largely distinct among species (Q1, Q2). Skate species in the North Atlantic and off South Africa have similar, patchy distribution patterns with little spatial overlap between dominant species [36], [68], [69]. When abundant species do overlap considerably in distribution, it is consistently reported among species of differing size. In the western GOA, the core regions of *B. aleutica* and *B. interrupta* overlapped substantially in Shelikof Strait, but early life stages of the larger species, *B. aleutica*, were absent. When a third species of intermediate size (*B. parmifera*) was present, its mode occurred between that of the other two congeners [70]. Off California, the core regions of *B. kincaidii* and *R. rhina* distribution overlapped, especially in association with headward parts of submarine canyons. *Raja rhina* grows to 1.80 meters [29], nearly three times the maximum size of *B. kincaidii* (66 cm TL) [71]. Among seven skates that constitute a skate assemblage between Nova Scotia and Cape Hatteras, North Carolina, most exhibited little spatial overlap; however, two species pairs (*Leucoraja erinacea*–*L. ocellata*; *Malacoraja senta*–*Amblyraja radiata*) had complementary distributions [72]. Each pair consisted of one species that attained a maximum size that was nearly twice that of the other [73]. A similar relationship has been demonstrated among demersal rockfish congeners in the ENP that are sympatric with *R. rhina* and *B. kincaidii*. *Sebastolobus alascanus* occupies shallower depths than similar sized *S. altivelis*, but moves down slope to shared habitats after attaining larger sizes [74].

Limited competition for prey resources may be a primary factor driving the observed co-occurrence of some skate species pairs. Diet composition of skates generally is consistent among size classes: small skates and early life stages of large species (<∼50 cm TL) consume amphipods, small decapods, and polychaetes; medium-sized skates (<∼100 cm TL) consume decapods with some small fishes; and large skates (≥100 cm TL) consume fishes and large decapods [75]. Dietary differences, therefore, may facilitate coexistence among skates of different sizes by limiting exploitative competition. For instance, diets of *R. rhina* >60 cm TL differed markedly from those of *B. kincaidii* collected in the same habitats off central California [39]. Intraspecific competition is typically more intense than interspecific competition because individuals within a species are more ecologically similar to those of other species. Intraspecific competition among similar sized individuals may therefore further structure the distribution and abundance of the studied skate populations. A detailed examination of dietary variability will be necessary for a complete assessment of resource utilization in ENP skate assemblages, and to determine the extent of niche differentiation within and among species.

Spatial variability was evident by life stage and season for some ENP skates, but transitional and adult male and female distributions were similar within species. Some studies have reported sympatric occurrences of juveniles and adults (e.g., [76]), whereas others report segregation (e.g., [77]). It has been further suggested that juvenile hard-nosed skates (*Raja* spp.) occur at shallower depths than adults, whereas the opposite situation is described for *Bathyraja* spp. [32], [33], [51]. The results of this study provided only limited support for the suggested ontogenetic differences in distribution (e.g., *B. binoculata* off California, *B. aleutica* in the western GOA), and in some cases an opposite trend was observed (e.g., *B. kincaidii* off California). Complex seasonal immigration and emigration patterns of juveniles and adults also have been reported for some species of skates, resulting in spatial segregation by sex and life stage [77], [78], [79], [80]. It is possible that seasonal migrations and intraspecific segregation patterns are more widespread than reported because of inconsistencies in the methods and timing of specimen collection. For instance, sex ratios obtained from a limited time period or during multiple seasons may be insufficient to reliably infer sexual segregation [36], [45]. In this study, seasonal variability in distribution and abundance was typical among Californian skates, suggesting seasonal movements and possible immigration/emigration from the study region. Adult and transitional individuals of all species in both regions displayed very similar distribution patterns between sexes. Since the primary surveys used in this study occurred during summer months, it is suggested that sexual segregation is not apparent among mature and maturing individuals at this time. However, year-round surveys, and (ideally) telemetry studies are necessary to resolve ambiguity in these and similar results to better understand intraspecific spatial relationships of skates throughout ontogeny.

Spatial analysis of ENP skates has yielded new insights into skate spatial associations and assemblage structure. Investigation of autocorrelation and clustering in ENP skate distributions enabled the determination of regions of aggregation and relative scarcity for skates within the larger seascape. Skate populations were highly clustered off central California and in the western GOA, with the distances of maximum peak clustering substantially greater for Alaskan species. This difference may be reflective of the greater relative abundance of California skates. Fishing pressure, environmental characteristics, geographic location and faunal composition differ substantially between study regions. The design and gear specifications of NMFS surveys also differed between study regions (e.g., [67], [81]), as did the relative size of the study regions. Therefore, any conclusions about the reasons for these perceived differences are largely speculative. Each assemblage contained five primary species of variable sizes. Differences in size composition are typical, but species richness in the study regions is depauperate compared to most continental shelf and upper continental slope assemblages [36], [72], [82]. In Alaska, several deep-water species venture above 600 m, and when these are considered, regional skate diversity is relatively high [38], [71]. However, off California only one such deepwater vagrant, *B. trachura*, is present. It is not known if this discrepancy in skate diversity between ENP regions is a result of tropical submergence in California skates or a result of variable benthic community structure between locations. As is typically noted, the assemblages contained endemics (i.e., *R. inornata*, *R. stellulata*, *B. parmifera*) as well as wide-ranging species (*B. binoculata*, *R. rhina, B. aleutica*) [7] [71]. *Bathyraja interrupta*, which was originally believed to range from the Bering Sea to southern California, appears to be a species complex consisting of *B. kincaidii* off the West Coast and potentially several Alaskan species (J. Orr, NMFS–AFSC; pers. comm.). The largest species were the most widespread, which may indicate increased mobility, broader environmental tolerances, or possibly competitive dominance [75], [83], [84].

Considerable advances have been made in understanding spatial relationships of skates and the processes that drive observed distribution patterns. Based on a review of the available literature, the primary process at work appears to be temperature; with depth and substrate association serving to further define habitat niches. Temperature differences were noted among ENP skate species, especially between *B. binoculata* populations, which inhabited much colder waters at comparable depths in the GOA. A similar situation was exhibited by *Leucoraja erinacea* populations in the North Atlantic [76], demonstrating that: 1) allopatric skate populations may exhibit highly variable temperature associations, and 2) some species may have extremely broad temperature tolerances that enable large potential distributions. Skate populations may be concentrated at temperatures that are known to be suboptimal for growth and maintenance even when other, more favorable temperature regimes are locally available [37]. Temperature also appears to be a major driving force in determining the structure and dynamics of skate assemblages [23], [78], [82]. The common skate, *D. batis*, and a recently discovered cryptic species have overlapping depth and substrate associations, but distributions are largely dictated by variable thermal limits, resulting in considerable interspecific spatial segregation [31]. Migrations to spawning grounds cause additional temporal variability in skate assemblage structure [80], [82]. Inter- and Intraspecific depth zonation was observed among ENP skates and is a common condition within skate assemblages (e.g., [36], [85]). Off California, observed seasonal differences in depth distribution likely tracked temporal variation in bottom temperatures. Skate assemblages, and constituent species, appear to exhibit different sediment associations that may further differentiate habitat niches among sympatric skates [82], [85], [86]. Off California, habitat associations differed substantially among all species, with *R. rhina* exhibiting the most general characteristics and having the most widespread distribution. Based on these results, spatial niche differentiation appears to be more pronounced in skates than previously reported [6], [7].

Advances in knowledge of ENP skate biology include resolved depth distributions, new information on substrate and temperature preferences, and the determination of spatial associations at a variety of scales. In Alaska, depth distributions were largely consistent with previously reported information [38], although the depth information was expanded from predictions of common depth ranges and depths of maximum occurrence. In California, where skate identification has been less reliable, considerable confusion in skate depth distributions was resolved. *Raja inornata* has been reported to 1600 m [71]. Results of this study, however, indicate that it is largely a shelf species that ranges to the upper slope, with a predicted maximum occurrence in waters <75 m. Sporadic occurrences of skates well beyond their normal depth range have been reported but a discontinuity of this magnitude can only result from identification error [72], [82]. Smaller discontinuities are evident for *B. binoculata* and *R. stellulata*, which are largely distributed on the inner and mid shelf but have maximum reported depths of 800 m and 732 m, respectively. Verified specimens are necessary to resolve potential misidentification of these species in deep water.

Depth information was used to create temperature profiles for each species, which represents the first such reported information for ENP skates and enables a more nuanced understanding of skate distributions and comparisons among systems. Habitat associations were determined for several California species for the first time. *Raja stellulata* was most commonly associated with rocky reef habitats. This species has dermal denticles on its ventral side, perhaps to protect it from abrasion in these regions, a phenomenon that could be more widespread and warrants further attention. *Raja rhina* was mainly distributed on mixed and, to a lesser extent, soft habitats at a variety of spatial scales, whereas *B. kincaidii* was largely observed on mud. Since habitat associations of most groundfishes are spatially consistent (e.g., [87]), *R. rhina* also can be expected to occupy mixed and soft habitats in the GOA. Direct substrate associations of *R. inornata* could not be determined, but its center of aggregation was largely restricted to the wide continental shelf region between Monterey and San Francisco. This region consists mainly of mud, with sands confined to inner and outer shelf waters [88]. All ENP skate species but the California population of *R. rhina* exhibited long-term areas of high aggregation. Instead, *R. rhina* distribution was generally ubiquitous from the mid shelf to upper slope off central California.

Skate management in the GOA has advanced to consider individual quotas for commonly exploited skate species (i.e., *B. binoculata*, *R. rhina*) and to establish a precautionary closure to directed fishing, but we offer some suggestions to enable continued progress. Habitat areas of particular concern (HAPCs), subsets of EFH that are considered to be vital to the long-term sustainability of particular species, have been established to protect six skate nursery grounds in the Bering Sea [32]. Results of hot spot analysis can be used to distinguish areas that are especially important to particular skate species, life stages, or the overall GOA assemblage, as a basis for the creation of HAPCs or to delineate fishery management areas and establish regional quotas. *Beringraja binoculata* is a large, nearshore species and therefore may be particularly vulnerable to fisheries exploitation [26]. Within two years of the inception of a *B. binoculata* fishery in Prince William Sound, a notable decline was observed in the overall abundance and relative proportion of subadult and adult females, contributing to the termination of the fishery (K. Goldman, ADFG, Homer; pers. comm.). By determining the common depth, temperature, and geographic distribution of this species, populations in the western GOA can be better monitored and managed. Detailed distribution and relative abundance information provided from this study also can inform food web models (e.g., [89]) by estimating the degree of spatial overlap among species, which may impact the intensity of their trophic interactions. Regional differences in the relative abundance of skates were noted among ADFG management regions. Length data should be analyzed to determine whether ontogenetic composition is consistent or variable among regions to better understand observed differences and possible causation. Survey data were grouped for the purposes of this study, but can be assessed on a yearly basis to determine how environmental variability may affect species composition and relative abundance between management regions.

Although the efficacy of California’s MPA experiment is still largely unknown, spatial management has been embraced along the West Coast and accurate, species specific spatial data are the foundation of this strategy [90]. Identification of skate landings remains a major problem in California, with over 99% of all such landings classified as “unspecified skate” [9]. *Raja inornata* is highly sexually dimorphic and may contain a cryptic species (*R. inornata inermis*) [28]. Based on recent NMFS–NWFSC surveys that record this species from the mid slope [67], it continues to be misidentified. Morphometric and genetic techniques should be combined to resolve any taxonomic issues with *R. inornata* and establish a reliable means of identification. The probability of a skate species occurring at a particular depth can be predicted from the regression models created for this project, and should be used to assess the reliability of extreme depth records. A synthesis of results regarding habitat associations, and depth and geographic distributions was used to update the HSP profile for adult *R. inornata*, which considerably reduced the amount and spatial distribution of highly suitable habitat regions. Since HSP profiles are used to distinguish EFH, the updated profile of *R. inornata* can directly inform the development of improved management plans for this species along the West Coast. Updated HSP profiles for *B. binoculata* and *R. rhina* also can be constructed from the results of this project for the same purpose.

Skates are a remarkably speciose group of fishes that are distributed throughout the world’s oceans. Project findings indicate that ENP skates: 1) occupy regions of differential importance (e.g., hot and cold spots) within the study regions, 2) are spatially segregated by species and often also by life stage, and 3) differ in size when they commonly co-occur. Skates therefore appear to have much more complex spatial associations than were previously described [6], [7]. Although they are morphologically conservative, skates may exhibit the greatest distribution of any fish group, occurring from pole to pole, at depths ranging from ≤2 m (*B. binoculata*, *R. stellulata*) [71] to 4156 m (*Rajella bigelowi*) [91]. Reliable identification, measurements, and life history research can facilitate relatively inexpensive, detailed spatial studies such as this one, which can provide a more nuanced understanding of skate distributions and assemblage structure. Seasonal surveys and telemetry studies are necessary to provide greater resolution to results of this project and to expand knowledge of skate migrations and habitat use. Coupled spatial and trophic studies can better assess the degree of niche differentiation among sympatric species and are an important next step in building a greater ecological understanding of skates. An improved ecological understanding of skates may in turn help explain their observed diversity patterns and can contribute to the formation of effective fishery management plans for benthic marine communities.
